# An international virtual hackathon to build tools for the analysis of structural variants within species ranging from coronaviruses to vertebrates

**DOI:** 10.12688/f1000research.51477.1

**Published:** 2021-03-26

**Authors:** Ann M. Mc Cartney, Medhat Mahmoud, Michael Jochum, Daniel Paiva Agustinho, Barry Zorman, Ahmad Al Khleifat, Fawaz Dabbaghie, Rupesh K Kesharwani, Moritz Smolka, Moez Dawood, Dreycey Albin, Elbay Aliyev, Hakeem Almabrazi, Ahmed Arslan, Advait Balaji, Sairam Behera, Kimberley Billingsley, Daniel L Cameron, Joyjit Daw, Eric T. Dawson, Wouter De Coster, Haowei Du, Christopher Dunn, Rocio Esteban, Angad Jolly, Divya Kalra, Chunxiao Liao, Yunxi Liu, Tsung-Yu Lu, James M Havrilla, Michael M Khayat, Maximillian Marin, Jean Monlong, Stephen Price, Alejandro Rafael Gener, Jingwen Ren, Sagayamary Sagayaradj, Nicolae Sapoval, Claude Sinner, Daniela C. Soto, Arda Soylev, Arun Subramaniyan, Najeeb Syed, Neha Tadimeti, Pamella Tater, Pankaj Vats, Justin Vaughn, Kimberly Walker, Gaojianyong Wang, Qiandong Zeng, Shangzhe Zhang, Tingting Zhao, Bryce Kille, Evan Biederstedt, Mark Chaisson, Adam English, Zev Kronenberg, Todd J. Treangen, Timothy Hefferon, Chen-Shan Chin, Ben Busby, Fritz J Sedlazeck

**Affiliations:** 1 NIH, Washington, USA; 2 Baylor College of Medicine, Houston, USA; 3 Washington University in St. Louis School of Medicine, St. Louis, USA; 4 King's College London, London, UK; 5 Institute for Medical Biometry and Bioinformatics, Düsseldorf, Germany; 6 University of Vienna, Vienna, Austria; 7 University of Colorado at Boulder, Boulder, USA; 8 Sidra Medicine, Doha, Qatar; 9 Stanford University School of Medicine, California, USA; 10 Rice University, Houston, USA; 11 Walter and Eliza Hall Institute of Medical Research, Melbourne, Australia; 12 NVIDIA Corporation, Santa Clara, California, USA; 13 VIB Center for Molecular Neurology, Antwerp, Belgium; 14 Pacific Biosciences, Menlo Park, USA; 15 Oxford Nanopore Technologies Ltd, Oxford, UK; 16 USC, Los Angeles, USA; 17 Children’s Hospital of Philadelphia, Philadelphia, USA; 18 Harvard Medical School, Boston, USA; 19 UC Santa Cruz Genomics Institute, Santa Cruz, USA; 20 Carnegie Mellon University, Pittsburgh, USA; 21 University of California, Davis, USA; 22 University of Texas at Dallas, Richardson, USA; 23 Konya Food and Agriculture University, Konya, Turkey; 24 University of Michigan, Ann Arbor, USA; 25 DNAnexus, Mountain View, USA; 26 USDA-ARS, Athens, USA; 27 Max Planck Institute for Molecular Genetics, Berlin, Germany; 28 Laboratory Corporation of America Holdings, Westborough, USA; 29 Lanzhou University, Lanzhou, China; 30 University of Pittsburgh, Pittsburgh, USA

**Keywords:** Structural variant, CNV, SARS-CoV-2, NextGeneration Sequencing

## Abstract

In October 2020, 62 scientists from nine nations worked together remotely in the Second Baylor College of Medicine & DNAnexus hackathon, focusing on different related topics on Structural Variation, Pan-genomes, and SARS-CoV-2 related research.

The overarching focus was to assess the current status of the field and identify the remaining challenges. Furthermore, how to combine the strengths of the different interests to drive research and method development forward. Over the four days, eight groups each designed and developed new open-source methods to improve the identification and analysis of variations among species, including humans and SARS-CoV-2. These included improvements in SV calling, genotyping, annotations and filtering. Together with advancements in benchmarking existing methods. Furthermore, groups focused on the diversity of SARS-CoV-2. Daily discussion summary and methods are available publicly at https://github.com/collaborativebioinformatics provides valuable insights for both participants and the research community.

## Introduction

Structural variants (SVs) comprise a number of genomic imbalances including copy number variations (CNVs), insertions (INS), deletions (DELs), inversions (INVs), duplications (DUPs), and inter-chromosomal translocations. ^1– 3^ SVs have been implicated as clinically significant mutations with proven associations to multiple diseases. ^4, 5^ Despite next-generation sequencing becoming increasingly common within the field of biomedical research, several practical challenges exist for comprehensively detecting and evaluating SVs particularly in regard to the high false positive or negative rate along with the accuracy of breakpoint prediction. ^6, 7^ While SV detection with genotyping arrays remains the most commonly used method, the toolbox for SV detection is expanding to incorporate the advancements in third generation sequencing technologies provided by Pacific BioSciences, ^8^ Oxford Nanopore Technologies, ^9, 10^ optical mapping and NanoString ^11^ to name a few. These advancements offer potential for solving previously unresolved structural variants.

In October 2020, 62 scientists from nine nations worked together remotely in the Second Baylor College of Medicine & DNAnexus hackathon, focusing on different related topics on SV, Pan-genomes, and SARS-CoV-2 related research. Consequently, this international structural variation hackathon meeting focused on eight themes: 1.) efficiently genotyping vast quantities of SVs; 2.) mapping CNVs to SV types; 3.) detecting and validating SVs for SARS-CoV-2; 4.) filtering high-confidence SV calls for clinical genomics; 5.) SV read-based phasing for haplotype analysis; 6.) genome graph generation without a reference; 7.) machine learning approaches to predict lab-of-origin of a sample.; and 8.) gene-centric data browsing for SV analysis.

Overall, this manuscript details our tools’ objectives, value-add, implementations, and applications and sets the foundation for further concept development beyond. In this article we present 10 software tools that were the results of this hackathon.

**NibbleSV: efficient genotyping of SVs from short read datasets.** Detection of SVs longer than a short-read (<500bp) DNA trace is very challenging as the SV allele becomes split across multiple reads. To this end, long read sequencing technology is preferential for overcoming this challenge however, although long read sequencing has proven more accurate in SV identification, obtaining accurate allele frequencies across a population is important in order to rank and identify potential pathogenic variations.

**Figure 1.**
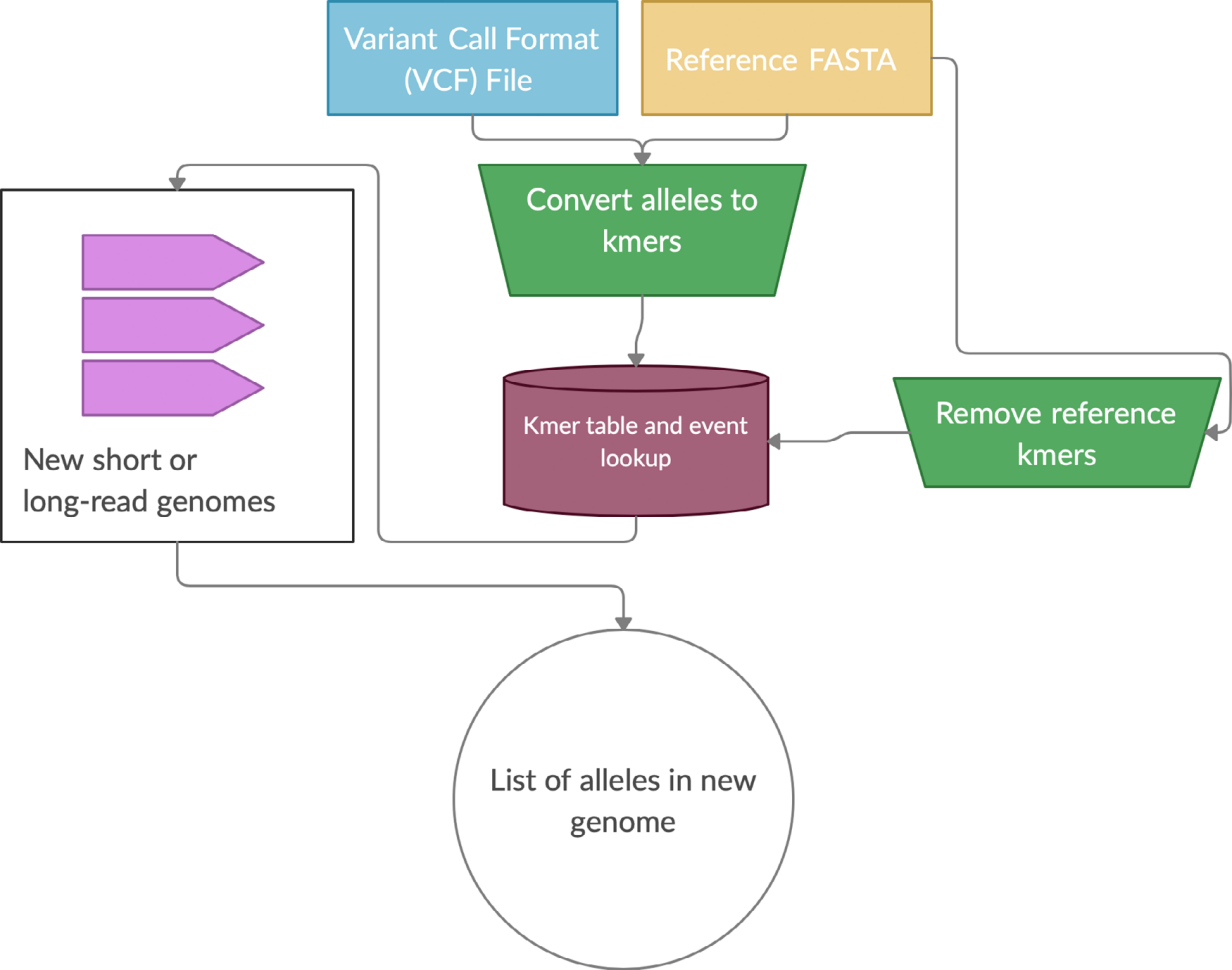
Overview of the NibbleSV software package workflow. Overview of NibbleSV workflow that utilises an input reference genome and file containing variant calls to generate a list of genotyped alleles using a kmer based strategy.

Thus, it is still important to genotype SV events in pre-existing short read datasets such as those provided by the 1000 genomes project, Topmed, CCDG, etc. Recently, two main approaches, Paragraph ^12^ and VG, ^13^ have achieved this with high accuracy even for insertion SV events. However, these methods are computationally expensive particularly when the number of SVs to be genotyped per sample increases. Furthermore, and maybe more crucially, both methods rely on precise breakpoints that do not change in other samples, an assumption that is potentially flawed particularly over repetitive regions. NibbleSV is a software package able to efficiently genotype vast quantities of SVs whilst also using a kmer catalogue of SVs in order to circumvent the need for re-mapping the same dataset to different versions of the same reference genome (e.g. hg19 vs. GRCH38 vs. CHM13), again aiding computational efficiency ( Figure 1).

**Figure 2.**
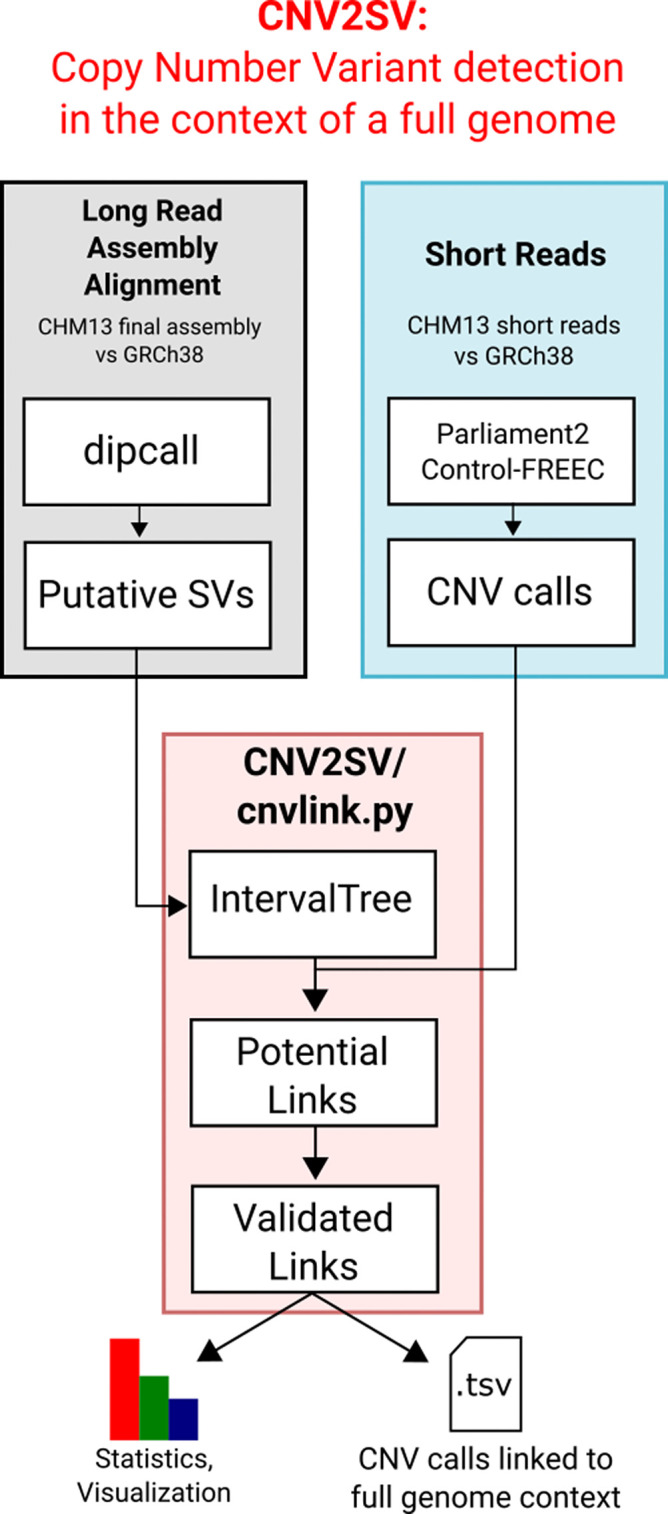
A graphical overview of the CNV2SV pipeline. CNV2SV software pipeline that utilises both short and long read data as input to calculate the frequency of copy number variants across complete genomes.

**CNV2SV: supplement CNV calling in SV detection.** CNVs are a subset of SV consisting of deletions and duplications. One common challenge is mapping CNVs to specific SV types. Automatically linking these events together will improve our understanding and interpretation of regions where large CNVs occur and potentially also lead to improvements in breakpoint accuracy and thus resolution if the breakpoints are more complex (i.e. including other SV events, e.g. inversions). Here, we demonstrate CNV2SV using the haploid human genome of T2T CHM13 and will showcase CNV2SV on additional diploid, publicly available samples ( Figure 2).

**CoronaSV: SV pipeline for SARS-CoV-2.** While deletions have been reported in several SARS-CoV-2 genomes at consensus level, ^14, 15^ the confidence in how these deletions are detected has not yet thoroughly been evaluated. Existing methods for detecting SV at the individual read level often suffer from false positive calls. ^16, 17^ Additionally, analyses with different variant calling pipelines often result in inconsistent calls. ^18, 19^ To examine the landscape and extent of SV across SARS-Cov-2 genomes, a method for generating accurate and trustworthy SV calls is needed. With this in mind, we developed the CoronaSV bioinformatics pipeline.

**Figure 3.**
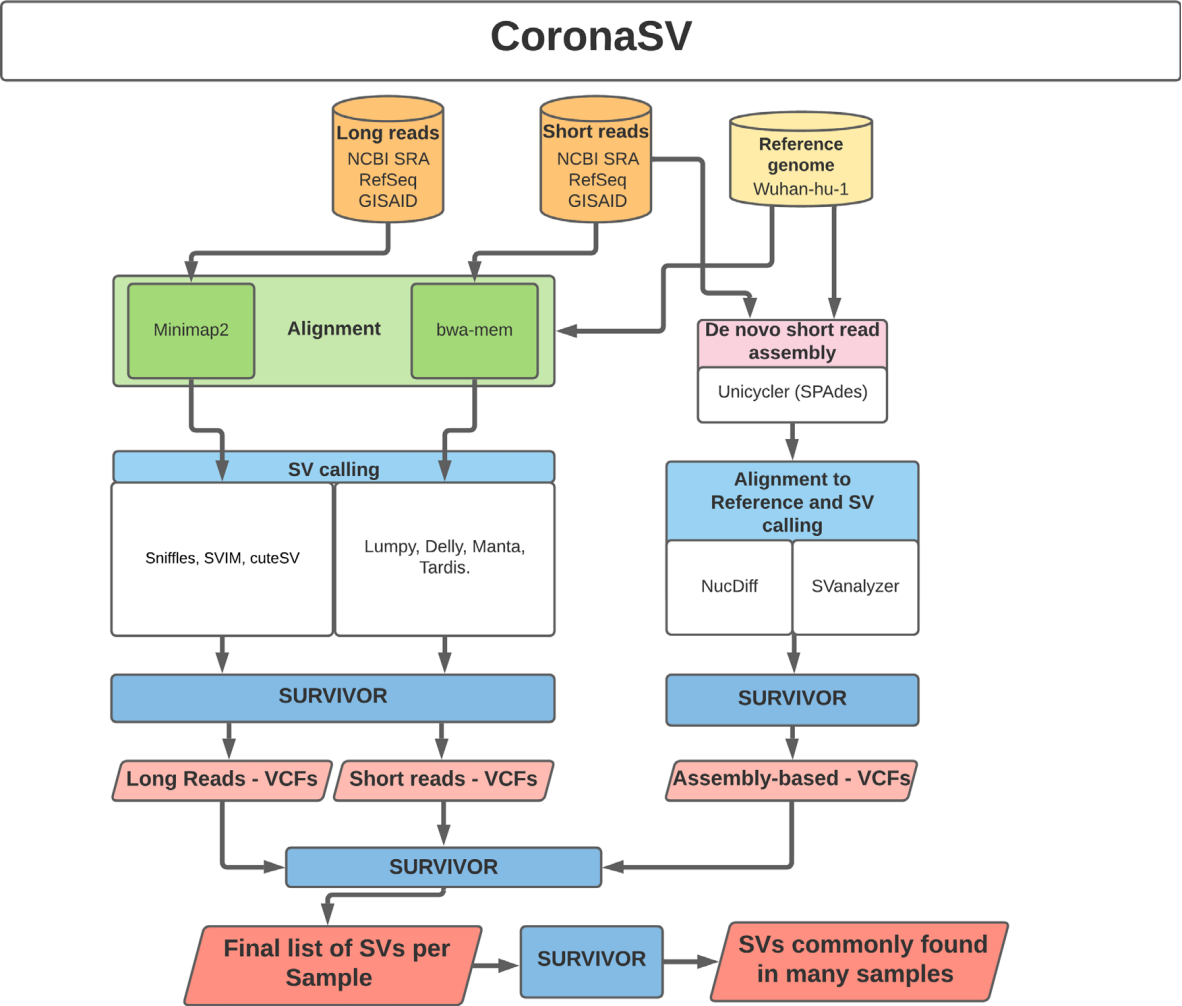
Illustration of CoronaSV software implementation. Illustration of the CoronaSV package workflow that takes SARS-CoV-2 short and long read data types along with a SARS-CoV-2 reference genome as input and generates a set of commonly found SVs.

CoronaSV is a SV detection and SV validation pipeline for SARS-CoV-2 that combines an ensemble SV calling approach that relies on both long read and short read sequencing technologies ( Figure 3). Both assembly-based and read based SV detection methods are used by CoronaSV. By combining different sequencing technologies and variant detection approaches, we can identify both a) confident SV call set and b) artifacts that may result from specific technologies + computational approaches.

**CleanSV: Filtering High-quality SVs.** Short-read sequencing is performed within clinical genomics to both inform and directly guide patient care. This has been immensely successful for various Mendelian disorders, where patients now routinely have their genomes sequenced to detect high-quality variants. Indeed, this approach has been utilized within clinical genomics for close to a decade, ^20^ often to correct misdiagnoses (see ^21^ for a recent example).

Within precision oncology, short-read sequencing (normally targeted sequencing or whole exome sequencing (WES)) has proved successful not only illuminating the nature of specific cancers, but also guiding novel drug development. Today routine sequencing is used to apply therapies for specific cancer subtypes, and influence the treatment of individual patients. ^22, 23^ For clinical work, samples from tumors are sequenced for somatic variants in well-studied oncogenes/tumor suppressor genes. Bioinformaticians will then manually investigate the variant calls within IGV in order to validate how accurate they are, and finally send reports summarizing these data to clinicians.

However, SV calling using short-read data is marred by high false positive (FP) call rates, sometimes up to 90% with modern callers. ^10, 24, 25^ As a result, manual curation for each patient proves oppressively time-consuming for the needs of modern precision care and is prone to human error. Even though aneuploidy has been long studied for its role in tumor progression (see ^26^ for a recent review), due to algorithmic uncertainties, routine inclusion of high-confidence SVs within clinical reports is often infeasible today.

Therefore, there exists a pressing need within bioinformatics to develop methods to remove false positives from the outputs commonly used SV callers, and benchmark their performance across a variety of assays (including a range of sequencing depths and tumor purities). Individual SV callers rely upon specific strategies to detect SVs, which makes the nature of the false positives algorithm-specific. Having access to a call set with a lower false positive rate would certainly not eliminate the requirements of manual curation, but it would make the problem more tractable.

The goal of this project was to develop a set of publicly available filters tailored for cancer genomics which have been measured to perform reliably across popular SV callers, as the filters must be specific to the SV caller used. Using a large cohort of high-quality normal whole genome sequencing (WGS) samples, we perform systematic false positive filtering. SVs labeled by the algorithm as somatic have evidence as actually being germline, while others are algorithmic artifacts. With such filters, bioinformaticians would have access to a set of high-quality somatic calls to manually curate, which could finally result in more robust clinical reports.

**Sniphles: Phasing SVs with parallel programming.** Phasing infers the correct cis or trans relationship between different heterozygous variations facilitating accurate haplotype reconstruction. ^27^ Protocols and programs utilizing molecular phasing (chromosomal separation at the bench before sequencing), pedigree-based phasing (matching parental and offspring genotypes to understand the haplotype), population-based phasing (using genotype data from large cohorts to infer haplotypes), and read-based phasing (mapping sequencing reads with the same variants to construct a haplotype) are all successful approaches to phasing next-generation sequencing data. ^28^ The long-reads of third generation sequencing have bolstered our ability to phase longer and more comprehensive haplotype blocks. ^29^ More comprehensive haplotype blocks increase our ability to accurately phase structural variants.

The goal of this project is to develop a wrapper script around the Sniffles SV caller ^10^ to properly phase SV and augment the ability of Sniffles to accurately call SV ( Figure 5). This result is obtained by using phased reads generated by SNV phasing tools such as WhatsHap or LongShot, ^27, 30^ and subsequently call SVs on the haploid phase blocks separately using temporary files before finally merging both haplotypes to obtain a single VCF file. As this algorithm processes each phase block separately this is attractive for parallelization. Our wrapper script additionally makes Sniphles compatible with alignments in the CRAM format.

**Swagg: Structural Variation With Annotated Graph Genomes (Swagg).** Most graphical approaches to variant calling only use genome graphs. While this information helps illustrate variation on a genomic level, it does not show variation on the individual protein level. To help leverage the power of graph approaches for SV calling, we introduce a pipeline that delivers both protein and genome graphs.

Swagg is a pipeline that enables the construction of genome graphs from read data ( Figure 6). The input into the pipeline is sequence reads with or without a reference genome(s). Reads can be short reads or preprocessed (basecalled) long reads. These reads are then assembled into longer contigs which are mapped back to the reference genome to highlight discrepancies with the reference genome. These discrepancies can be caused by real mutations or sequencing artifacts and easily identified using SV tools, which output VCF files for each read set. These VCF files are taken together to make the genome graph at the end of the pipeline. The overall pipeline and intertwined modules are shown below. In addition to the pipeline for creating graph genome and graph proteins, we also have a module for simulating reads based on an input reference genome.

**PanOriginSV: detecting lab-of-origin.** The advent of novel synthetic biology methods and organic bench-top synthesis toolkits like CRISPR ^31^ has enabled rapid developments in genetic engineering. However, this progress has also introduced biosafety concerns surrounding the intentional or unintentional misuse of these tools. In order to increase accountability, the lab-of-origin studies attempt to map a set of plasmids to their lab-of-origin. Subsequently, the Genetic Engineering Attribution Challenge (GEAC) was announced, inviting open source tools from the community that could best predict the lab-of-origin.

Previous methods have employed machine learning or deep learning-based approaches that despite their promise, suffer from sub-optimal accuracy, long training times as well as explainability issues. Recently, a new alignment based tool *PlasmidHawk* ^32^ reported higher accuracy than machine learning tools. *PlasmidHawk* relies on linear pangenome constructs to align query sequences to a pangenome in order to best determine the Top-1 and Top-10 candidate labs. Though *PlasmidHawk* has a higher accuracy, the runtimes to create the linear plasmid are non-scalable to larger datasets. Another drawback being the linear pangenome doesn’t incorporate SV, which could be important to predict hard-to-classify sequences. To address some of these challenges, we propose a tool PanOriginSV that combines machine learning approaches with graphical pangenome based alignment to predict lab-of-origin ( Figure 7).

PanOriginSV creates multiple pangenome graphs from similar training sequences using BCALM ^33^ creating a variation graph that incorporates SV and aligns the sequences back to the graph using GraphAligner. ^34^ The most similar training sequences for graph construction are clustered using MMSEQ2. ^33, 35^ After this, top alignments, scores to the pangenome and sequence metadata are considered as features for a downstream machine learning model towards lab-of-origin prediction.

**GeneVar: SV Browser**. Next-generation sequencing provides the ability to sequence extended genomic regions or a whole-genome relatively cheaply and rapidly, making it a powerful technique to uncover the genetic architecture of diseases. However, significant challenges remain, including interpreting and prioritizing the identified variants and setting up the appropriate analysis pipeline to cover the necessary spectrum of genetic factors, which includes expansions, repeats, insertions/deletions (indels), SV and point mutations. For those outside the immediate field of genetics, a group that includes researchers, hospital staff, general practitioners, and increasingly, patients who have paid to have their genome sequenced privately, the interpretation of findings is particularly challenging. Although various tools are available to predict the pathogenicity of a protein-changing variant, they do not always agree, further compounding the problem. Furthermore, with the increasing availability of next-generation sequencing data, non-specialists, including health care professionals and patients, are obtaining their genomic information without a corresponding ability to analyse and interpret it as the relevance of novel or existing variants in genes is not always apparent. Similarly SV analysis ^36, 37^ and its interpretation requires care in regard to sample and platform selection, quality control, statistical analysis, results prioritisation, and replication strategy.

Here we present GeneVar, an open access, gene centric data browser for SV analysis ( Figure 8). GeneVar takes as input a gene name or ID and produces a report that informs the user of all SVs overlapping the gene and any non-coding regulatory elements affecting expression of the gene. The tool is intended to have a clinical focus, informing the interpretation of SV pertaining to a gene name provided by the user.

**SVTeaser: simulated data for SV benchmarking.** SV detection tools often have a large number of wrongly detected variations ^16, 24^ requiring benchmarking to assess method quality before finalizing a workflow. Few tools are currently available to simulate data for SV benchmarking. SVTeaser is a tool for rapid assessment of SV calling fidelity with two main use-cases: 1) genotyping a set of pre-ascertained SVs and 2) benchmarking a new algorithm against pre-existing tools across a range of parameters. Users simply supply SVTeaser with a reference sequence file (.fasta) and, optionally, a set of SVs (.vcf). SVTeaser outputs simulated reads across a range of read lengths and depths and provides a downstream dataframe based analysis framework for evaluating accuracy ( Figure 9). SVTeaser achieves rapid assessment by downsampling the full reference to a subset of numerous 10kb samples to which it adds SVs.

**XSVLen: haplotype-resolved assemblies for benchmarking SVs.** Since the development of a “gold standard” SV set, sequencing technologies and assembly algorithms have improved to enable nearly complete haplotype-resolved assemblies of human genomes. XSVLen is a framework ( Figure 10) to use haplotype-resolved assemblies for benchmarking SV detection algorithms. Each variant call may be considered an operation to be applied to the reference genome. Our framework for benchmarking SV callsets is to apply SV operations to the reference genome and compare the modified reference against the haplotype-resolved assemblies. This approach allows for SV calls that are different but produce similar sequences due to the repetitive nature of the genome to be scored as valid. In this manner, all variants in a region that is accurately assembled in both haplotypes may be benchmarked using this approach. We demonstrate the effectiveness of this approach by scoring SV calls generated from Oxford Nanopore reads on the HG002 genome ^38^ using CuteSV ^39^ and comparing against gold-standard calls by Truvari ( https://github.com/spiralgenetics/truvari). This approach can be extended to use any haplotype-resolved assembly to benchmark SV callsets in additional genomes, enabling benchmarks as a distribution across call sets.

## Methods

### Implementation

**NibbleSV:** NibbleSV is a lightweight, scalable and easy to apply method to identify the frequency of SV events across short read data sets. As such, NibbleSV extracts kmers that are informative if an SV is present or if an SV is absent given the breakpoints of the previous predicted SV. Subsequently, NibbleSV scans the short read bam or fastq file for the presence of these k-mers and counts their number of occurrences. In the end, NibbleSV extends the VCF file with tags holding information about the number of times an SV is supported by kmers or not ( Figure 1).

**CNV2SV:** CNV2SV is a tool developed to identify CNVs in the context of a whole genome sequence ( Figure 2). CNV2SV requires three input files which is a BED or VCF formats (from Parliament2, ^40^ Control-FREEC, ^41^ a putative SVs from genome-genome alignment (vcf format, from dipcall) and assembled reference files (fasta files) from both the reference and the assembled sample. In addition, we require preinstalled packages such as intervaltree, mappy, pyfaidx together with python v.3.8.* or higher. The visualisation for CNV2SV requires the installment of R together with the circularlize package, matplotlib, seaborn and pandas. CNV2SV currently relies on an installation of Control-FREEC which is needed for the CNV calling from short reads. The main output of CNV2SV (cnvlink.py) comprises the best matching linked SV for each CNV call, with summary statistics including alignment overlap, mismatches, respective start and end positions useful for evaluating e.g. the quality/resolution of breakpoints identified by the CNV callers. Furthermore, all additionally identified adjacent and distant SVs are reported separately for each CNV. The raw output can be further visualized to show the CNV-SV links identified across the genome in a circular plot, as well as summary statistics for the linking results ( Figure 2).

**CoronaSV:** CoronaSV is a method developed for generating accurate and trustworthy SV calls across SARS-Cov-2 genomes. The tool utilises available SRA data from SARS-CoV-2 isolates that have been sequenced with both Illumina and ONT platforms. CoronaSV utilizes a combination of three different approaches: read-based SV detection with paired-end Illumina reads and ONT long-reads, as well as assembly-based SV detection using both short and long-reads ( Figure 3). All the software packages used by CoronaSV can be installed via the Conda package manager ( https://github.com/conda/conda).

**Figure 4.**
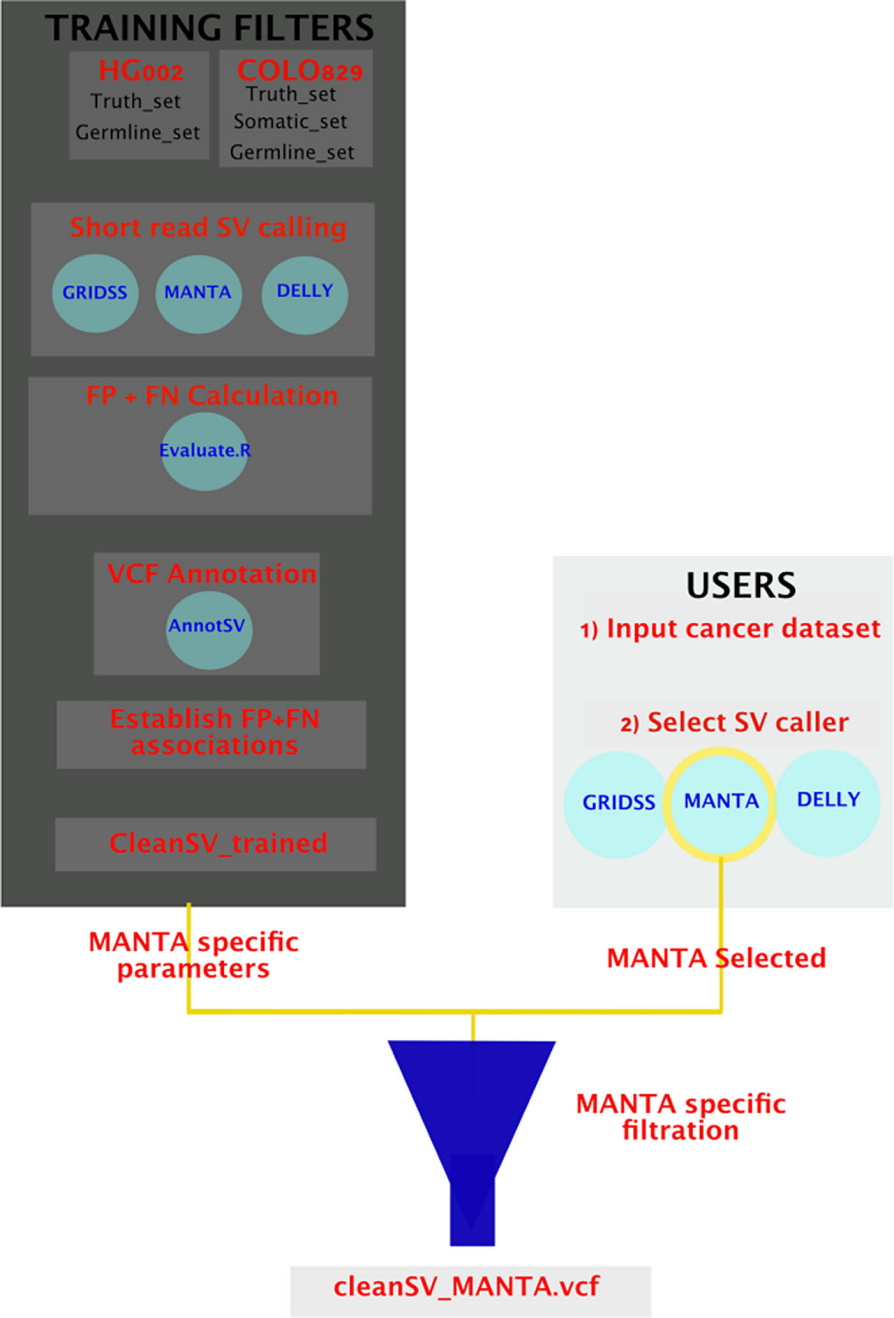
An illustration of the approach used by CleanSV to generate and implement filtration of SV calls. CleanSV pipeline highlighting methods used to generate adequate filters that can be utilised by clinicians to filter false positive and mislabeled SV calls from short-read cancer datasets.

**CleanSV****:** The goal of the hackathon project was to develop filters and QC checks to remove false positive calls from common SV callers. Currently, within clinical genomics, it’s exceptionally difficult to categorize true positives from false positives, thus making accurate diagnoses virtually impossible. The situation is even more complicated within clinical oncology, as researchers need to precisely separate true somatic calls from false positives and (potential) germline calls. In order to aid with precision SV calling, the team wrote a set of scripts to be used with short-read SV callers, allowing researchers to better generate a set of high-quality SVs to further investigate manually ( Figure 4).

For cancer genomics, groups normally develop in-house filters to improve the precision of SV calling. The scripts developed for this project accept as input GRIDSS, ^42^ Manta, ^43^ DELLY, ^44^ and SvABA ^45^ calls from short-read WGS data, along with a manually-curated reference set of calls designated as ground truth. The reference set was curated using a paired melanoma and normal lymphoblastoid COLO829 cell lines using four different technologies (Illumina HiSeq, Oxford Nanopore, Pacific Biosciences and 10x Genomics), along with extensive external validation. ^46^ Using the reference set, we proceeded to investigate the presence and nature of false positives from the initial callsets. (Note that we focused on WGS for this hackathon, but a similar approach could be applied to other assays such as WES.) Samplot ^47^ visualization of read data allowed manual curation of parameters associated with FP calls, and associations between AnnotSV ^47, 48^ annotated parameters and FPR helped identify additional FP-associated SV parameters ( Figure 4). Along with the manually-curated reference set, the panel of normal (PON) used for further filtering was generated from a compiled set of high-quality germline calls using 3,782 normal samples freshly-sequenced at a median depth of 38x by the Hartwig Medical Foundation. ^49, 50^

**Figure 5.**
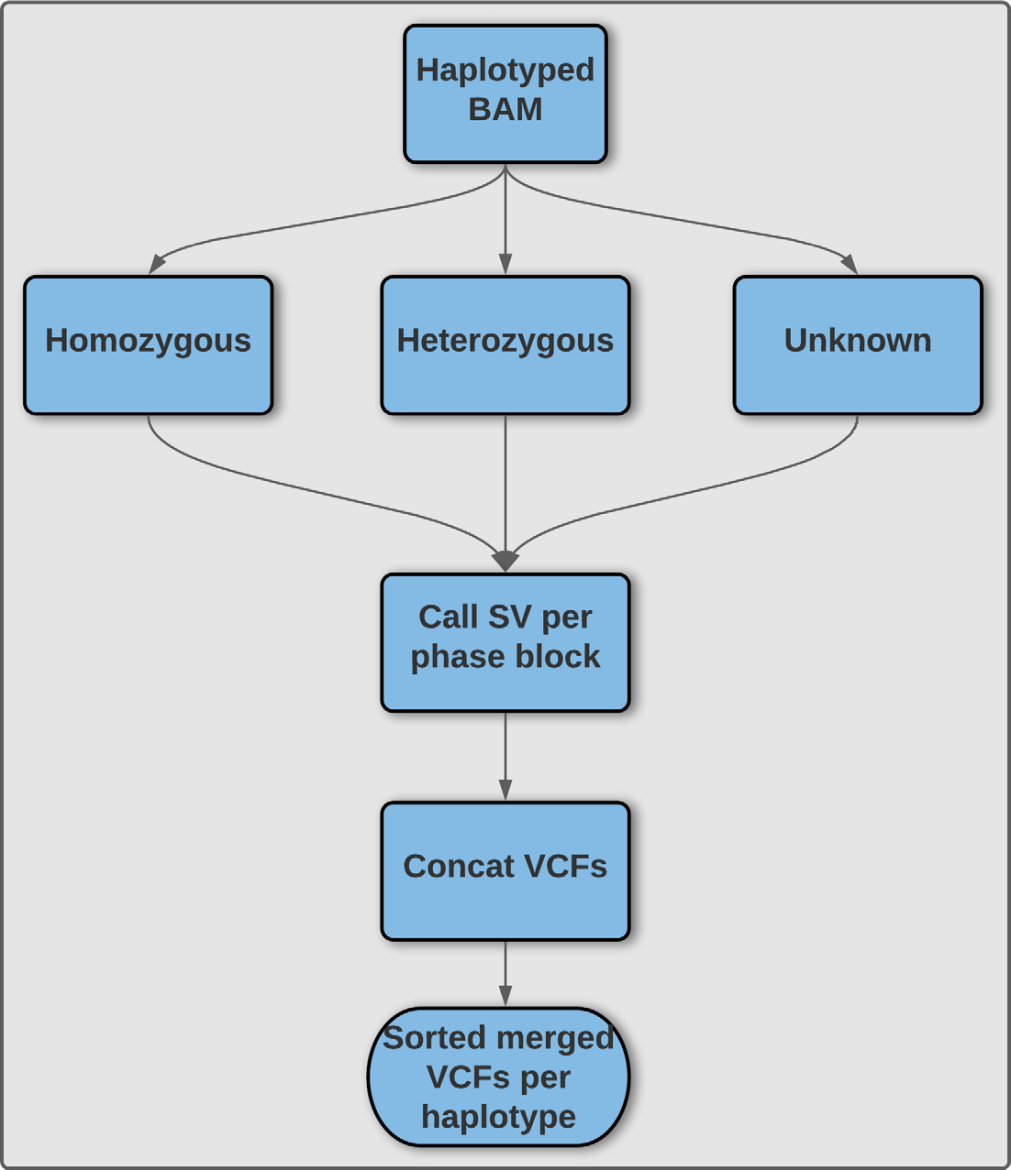
**Illustration of methodology utilized by Sniphles to produce a phased structural variant call set.** An overview of the Sniphles pipeline demonstrating how a haplotyped input bam file is used to generate a phased structural variant call set.

**Sniphles:** The main idea is to phase the identified SVs. We use two approaches; the first is to extract the tagged reads from the bam file and use these reads to phase the SVs if not conflicted. The second approach is to split the haplotype bam file based on the haplotype tag, using each split bam file to call SVs separately, this method called (Sniphles). Sniphles utilize information impeded in haplotypes, bam file and reads info support SVs. This method phases structural variants and augments the ability of Sniphles to accurately call SVs ( Figure 5). Sniphles is implemented in Python 3, and it takes a haplotyped bam, and a SV VCF file as input and produces a phased vcf file as output.

**Figure 6.**
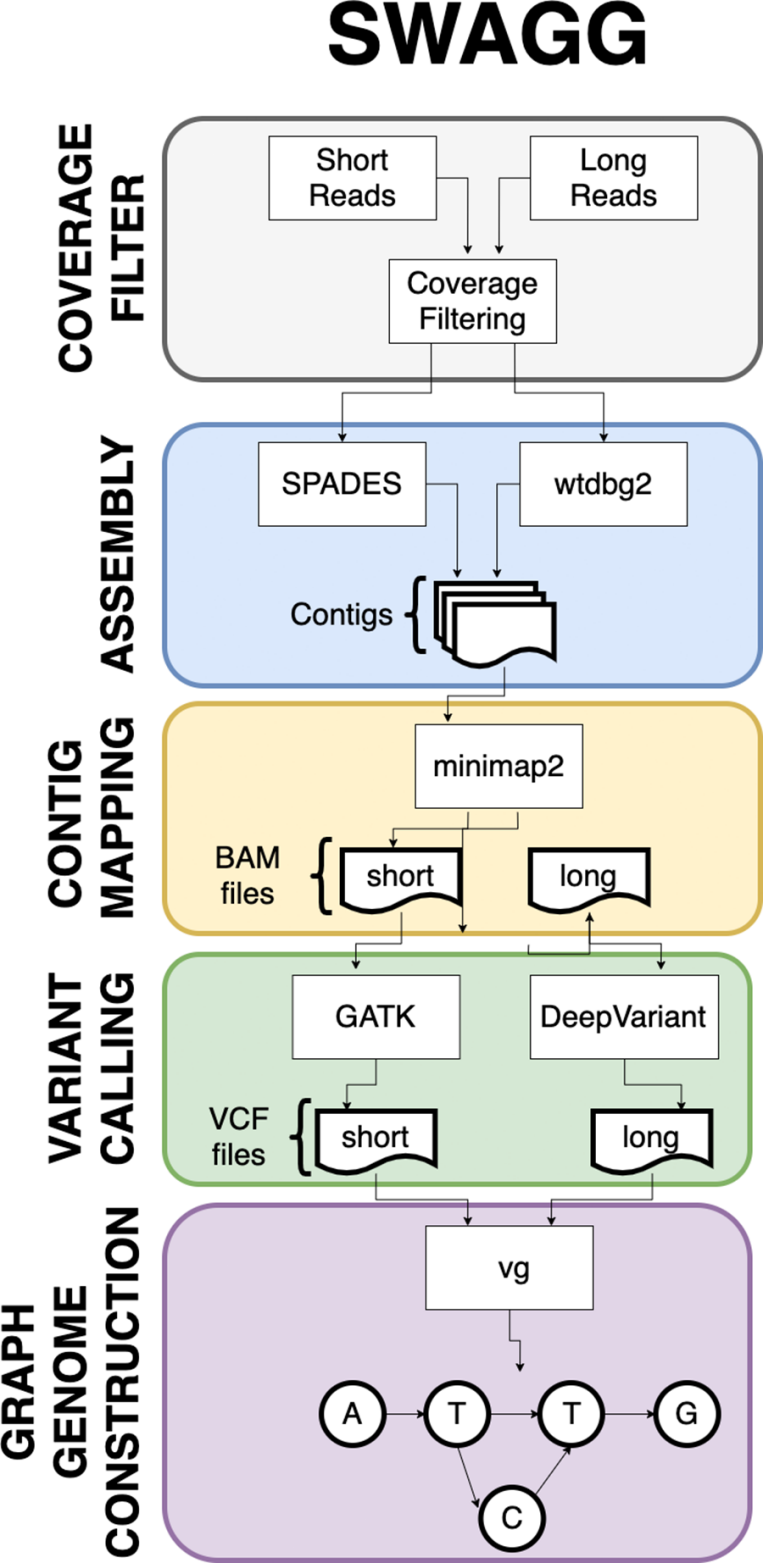
Outline of the SWAGG software package workflow. Illustration of the SWAGG pipeline that utilises both long and short read datasets for the construction of graph genomes.

**Swagg**: Structural Variation with Annotated Graph Genomes (SWAGG) is a pipeline to make genome graphs from read data. The input into the pipeline is reads either with or without reference genome(s). Reads can be short-reads or preprocessed (basecalled) long-reads. Reads are assembled into longer contigs, and contigs are mapped back to the reference genome to look for discrepancies with the reference genome. These discrepancies can be either real mutations or sequencing artifacts, and are found using structural variant tools which output VCF files for each read set. These VCF files are taken together to make the genome graph at the end of the pipeline ( Figure 6).

**Figure 7.**
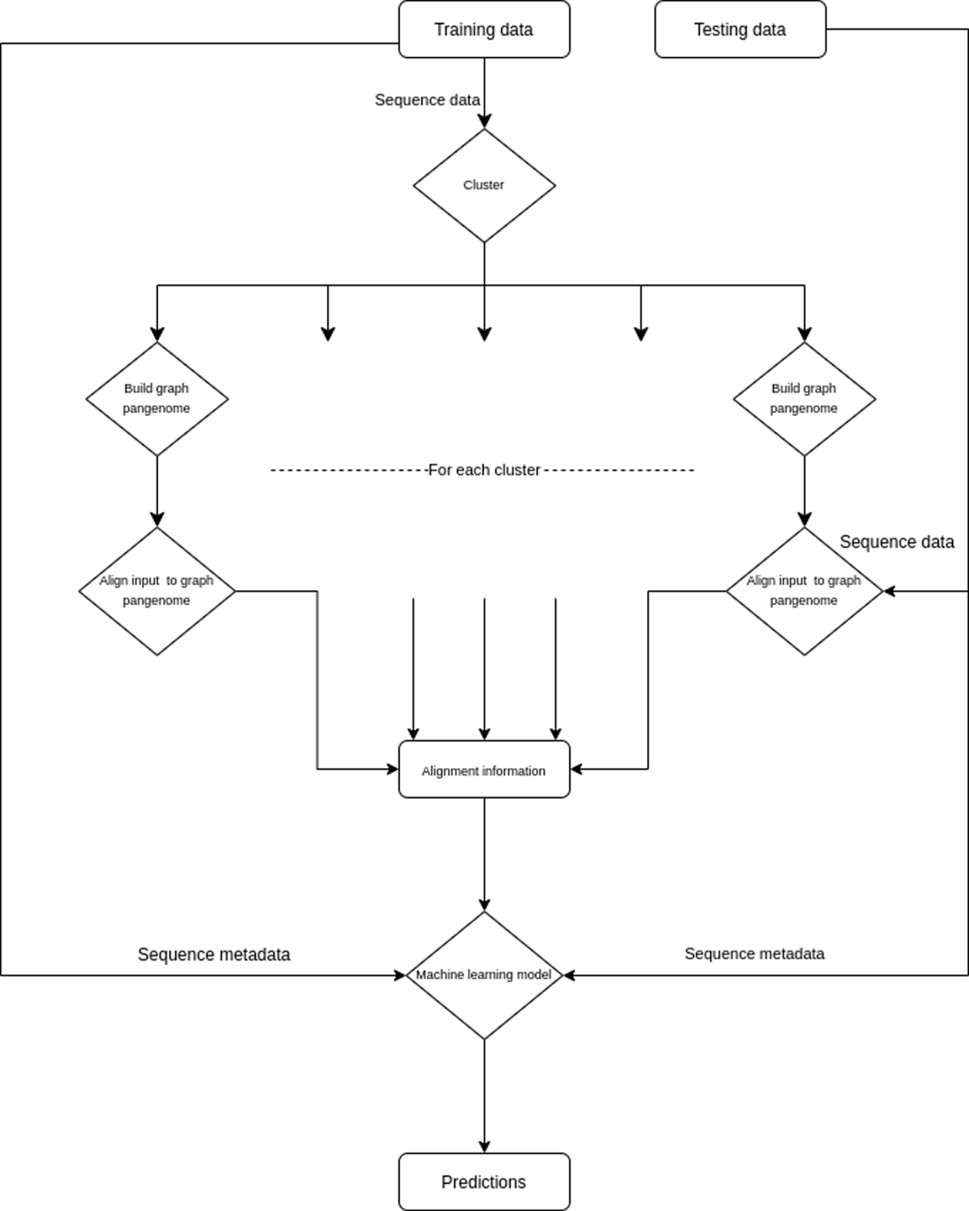
Implementation strategy of the PanOriginSV software package. Pipeline demonstrating the PanOriginSV software package that’s implementation determines that lab-of-origin input sequencing data.

**PanOriginSV:** This tool is a lab-of-origin prediction tool that combines machine learning approaches with graphical pangenome based alignment to predict lab-of-origin ( Figure 7). PanOriginSV is implemented in Python 3 and uses the scikit-learn package for deploying machine learning models. PanOriginSV also relies on MMSEQ2 for clustering, BCALM for graph construction and minigraph for graph alignment. Given a training set of engineered plasmids and their source labs, this software can predict the lab of origin of a test set of plasmids.

**Figure 8.**
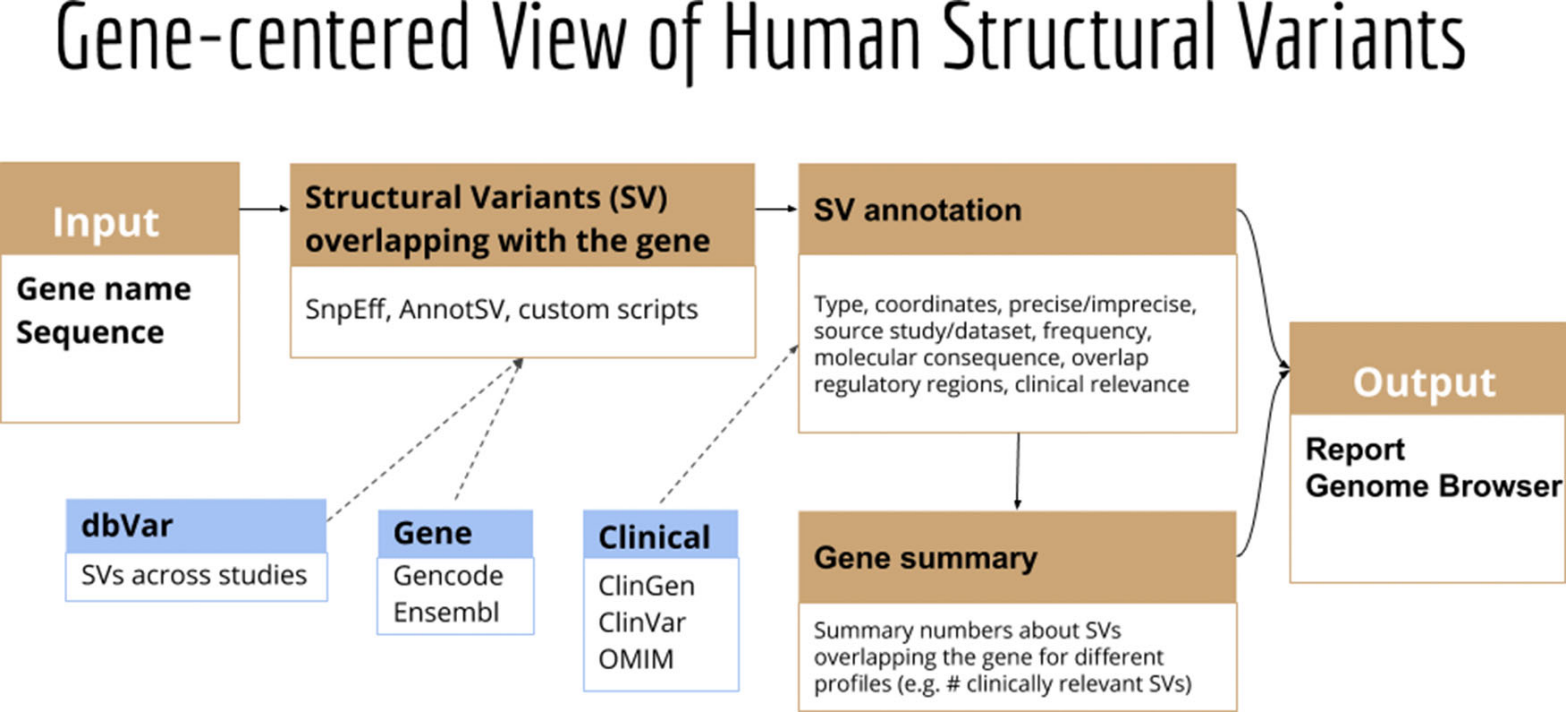
Methodology used by GeneVar for the production of summary report made available through genome browser. A graphical representation of the pipeline used by GeneVar in order to provide clinicians with a comprehensive summary of SVs associated with a user provided gene name.

**GeneVar**: The GeneVar tool was developed to help inform the clinical interpretation of structural variants pertaining to a user-provided gene. This software is an open access, gene-centric data browser for SV analysis. GeneVar is a web page application ( Figure 8). After entering the gene name (HGNC, Ensembl gene (ENSG), or transcript (ENST) identifier) in the search box on the homepage, the user is directed to the summary of the gene-specific page. GeneVar is available on GitHub ( https://github.com/collaborativebioinformatics/GeneVar). The repository provides detailed instructions for tool usage and installation. A bash script for automated installation of the required dependencies is also provided.

**Figure 9.**
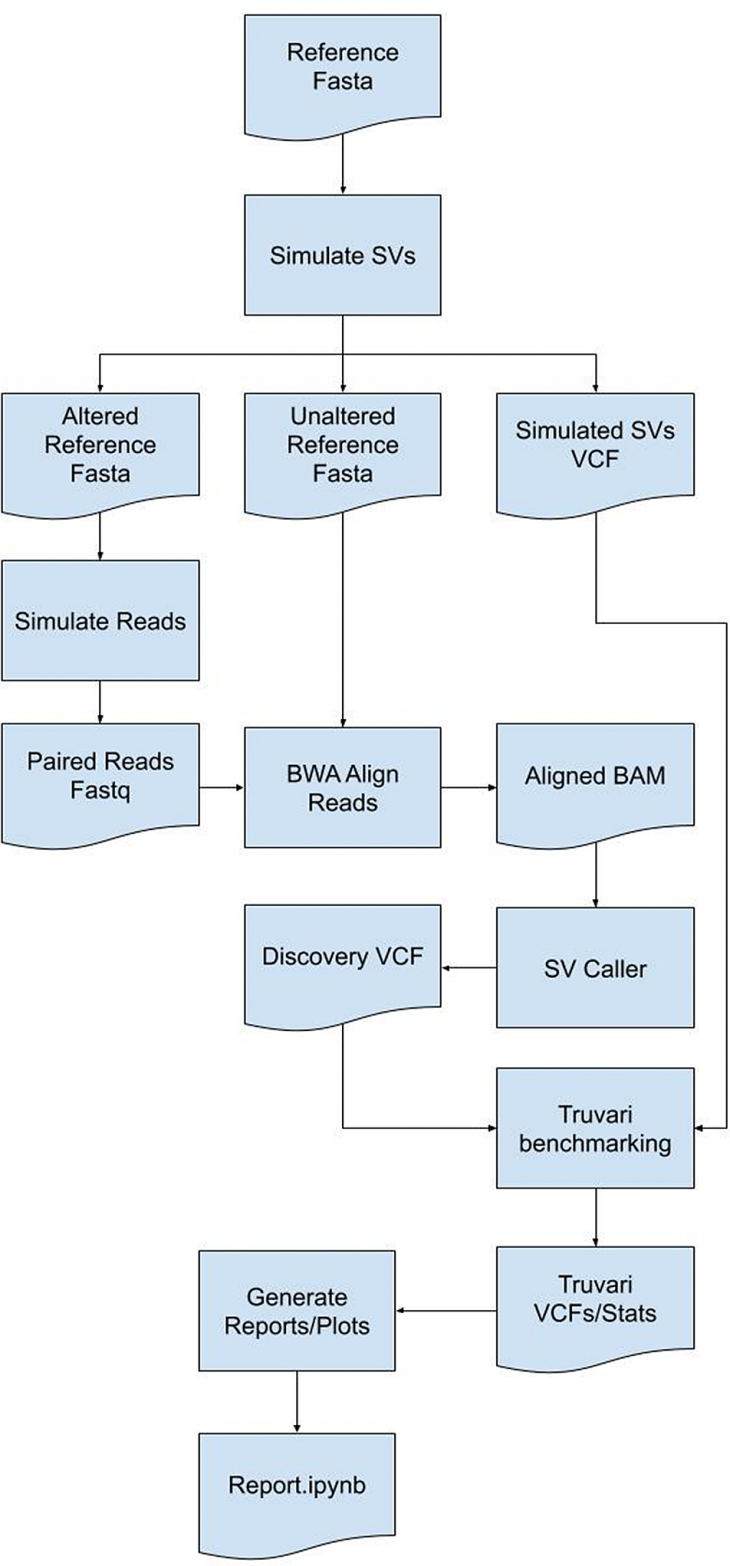
An illustration of the methodology implemented by the SVTeaser software package. SVTeaser software implementation showing how by using a reference genome a set of SVs can be simulated to benchmark SV calling tools to inform experimental design decisions prior to analyses.

**SVTeaser:** SVTeaser is a tool for rapid assessment of SV call fidelity created for geneticists designing experiments to genotype a set of pre-ascertained SVs and bioinformaticians benchmarking a new algorithm against pre-existing tools across a range of parameters ( Figure 9). Users are required to supply SVTeaser with a reference sequence file (.fasta) and, optionally, a set of SVs (.vcf). SVTeaser outputs assorted statistical metrics across a range of read lengths and depths. SVTeaser achieves rapid assessment by downsampling the full reference to a subset of numerous 10kb samples to which it adds SVs.

**Figure 10.**
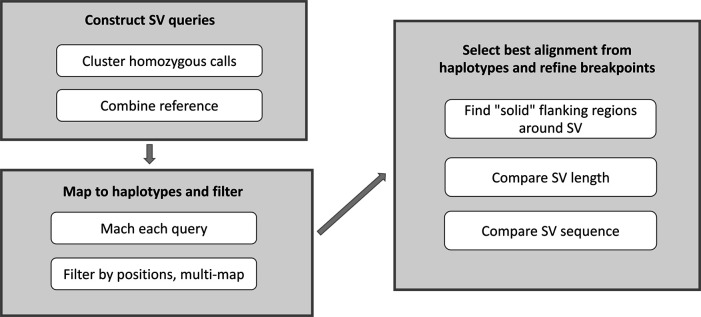
Implementation pipeline of XSVLen software package. A graphical representation of the XSVLen software pipeline showing the utilisation of haplotype-resolved de novo assembly and a VCF file to benchmarking SV detection algorithms.

**XSVLen:** This software is a framework for haplotype-resolved assemblies for benchmarking SV detection algorithms ( Figure 10). XSVLen takes a cuteSV or Sniffles ^10^ VCF output file and using reference coordinates will produce modified sequences having included inserted sequences or deleted sequences within the reference sequence. By creating these modified sequences, we could check for the presence of the predicted variants in haplotype-resolved assemblies. All methods are open-source licensed and have been made available on GitHub: https://github.com/collaborativebioinformatics.

### Operation

**NibbleSV:** NibbleSV requires a reference genome and VCF file that includes all the SV that should be genotyped ( Figure 1). Next, allele kmers for the reference and alternative are extracted. The extraction process includes each site’s flanking regions. Subsequently, the occurrence of these k-mers in the reference fasta file are counted. This step is necessary to prevent k-mer miscounting between reference vs. alternative allele. To enable scaling of NibbleSV for large data sets, the results of these two steps are written into a temporary file, which is all that is needed for the actual genotyping step. During the genotyping step, NibbleSV uses this small temporary file and the bam/cram file of the sample and identifies the presence/absence of the reference and alternative k-mer across the entire sample. This is very fast and requires only minimal resources of memory as the number of k-mers is limited. On completion of NibbleSV a scanning of the bam/cram file is carried out reporting which SV have been re-identified by adding a tag in the output VCF file of this sample ( Figure 1). The VCF per sample can then be merged to obtain population frequencies. The VCF per sample can then be merged to obtain population frequencies. NibbleSV requires 4Gb of memory, a single core and around 2GB hard disk space to store its index from e.g. GIAB HG002.

**CNV2SV****:** An overview of the CNV2SV workflow is illustrated in Figure 2 **.** CNV/SV are called from short read datasets using Parliament. ^51, 52^ Locations of duplications are determined using CNVnator ^53^ individual calls and combined calls. Copy number estimation for regions across the genomes is implemented by Control-FREEC that is converted into BED and VCF files. The genomes to be compared are aligned using dipcall,dot plots of the alignment constructed to identify potential regions of interest and extracted to VCF output. Calls for both short read and assembly based CNV/SV calls are merged to locate regions in which the duplication events overlap. Visualization scripts will be made available in future versions. Here, each called CNV from the short read dataset is compared to SVs identified from the genome-genome alignment. This is achieved in two steps: First, CNVs are queried against an interval tree structure containing the SVs from the genome-genome alignment to find adjacent CNV-SV pairs (<1000bp apart, putative tandem duplication events). For CNVs for which no matching SV can be identified in this way, the search is then extended to the whole genome (putative translocation events). All potential CNV-SV links are evaluated by sequence alignment using mappy (Python binding for minimap2, ^54^ https://pypi.org/project/mappy/, with a standard sequence identity threshold of 0.8. A quick-start example using CHM13 and GRCh38 data is available on our GitHub page. In addition, we hosted a detailed description of the output data on the GitHub page.

System requirements (see GitHub for more information): CNV2SV has been tested to work on a desktop system on the CHM13 data set with an Intel® i7-6700K Processor (4.00Ghz quad-core), 32GB RAM (less may be required), 50GB free disk space and running Unix-like operating system (e.g. Ubuntu-based distribution) or Windows subsystem for Linux running Ubuntu. The initial genome-genome alignment (CHM13 vs GRCh38) was computed on a cloud-based platform (DNANexus). CNV2SV requires Python (3.8 or newer). A full list of package dependencies is available on the GitHub page.

**CoronaSV:** All software packages used by CoronaSV can be installed via the Conda package manager. Additionally, the CoronaSV workflow is defined using Snakemake. Running the CoronaSV.smk snakemake pipeline handles downloading all specified data and processing of sequencing data to variant calls. Each step of the CoronaSV pipeline ( Figure 3) has a defined conda environment with exact versions of software specified for easy installation. CoronaSV utilizes three approaches that includes 1) read-based SV detection with paired-end Illumina reads, 2) ONT long-reads and 3) assembly-based SV detection. Illumina paired-end short-reads are trimmed using trimmomatic ^55^ and mapped to the SARS-CoV-2 reference using bwa mem. ^56^ After mapping, PCR duplicates are removed with Picard MarkDuplicates ( http://broadinstitute.github.io/picard). Structural variants are identified then using Delly, ^44^ Manta, ^43^ Lumpy, ^57^ and Tardis. ^58^ Nanopore long-reads are filtered using Nanofilt and mapped to SARS-CoV-2 reference using minimap2 with default parameters. SVs are then called using Sniffles, SVIM, and CuteSV. Read quality assessment is carried out by NanoPlot. In order to integrate assembly based methods, de novo SARS-CoV-2 assemblies were generated using Unicycler for short-read sequencing and Flye for ONT long-reads. NucDiff and SVanalyzer tools are used for assembly-to-assembly comparisons. Followup comparative analyses across callsets is implemented by SURVIVOR ^59^ ( Figure 3).

System requirements: CoronaSV is tested on Linux-based systems with multiple illumina and nanopore sequencing data (see GitHub for full list of the testing data). The RAM usage of CoronaSV depends on the size of input data. Peak RAM usage appears during de novo assembly using Unicycler, and 16 GB of RAM is sufficient for the pipeline to run on 8 CPU cores with additional 50Gb of disk space. CoronaSV requires python (version 3.6 or newer) and snakemake. Required tools and package dependencies can be found on GitHub page.

**CleanSV****:** The methods adopted to construct the CleanSVs filtration protocols are shown in Figure 4. In order to generate the data required to develop adequate filters, structural variants (SVs) were called on Illumina short reads using novoalign hs37d5 HG002 BAM ( ftp://ftp.1000genomes.ebi.ac.uk/vol1/ftp/technical/reference/phase2_reference_assembly_sequence/hs37d5.fa.gz) and Illumina short-read HiSeqX Ten hg19 COLO829 BAM ( https://nextcloud.hartwigmedicalfoundation.nl/s/LTiKTd8XxBqwaiC?path=%2FHMFTools-Resources%2FGRIDSS-Purple-Linx-Docke r) using GRIDSS, ^42^ Delly, ^44^ and Manta. ^43^ In HG002, the SV truthset (6) was used to determine the false positive (FP) SV calls in the short read dataset. Any SV calls that were found outside of the truthset Tier1 bed regions were then filtered. In the sample COLO829, the SV truthset (2) was used to determine the FP SV calls in the short-read dataset. Calls were inspected through manual curation by samplot (7). Generated samplots were annotated with UCSC table browser repeat tracks (8) and converted using vcfanno (9) as well as GC content by bedops file conversion from wig to bed (10).

We then compiled a set of high-quality germline calls using 3,782 normal samples freshly-sequenced at a median depth of 38x by the Hartwig Medical Foundation. ^49, 50^ We intiial hypothesized that such a large cohort could be used to both perform systematic FP filtering and possibly detect calls simply incorrectly labeled as somatic. The calls were filtered if a match within 2bp of the breakpoint was found in the PON. System requirements: The scripts to filter SV calls with either VCF or BEDPE format require R version 3.6.0 or higher, which is available for Linux, Mac OS, and Windows. The analyses within were run on R version 4.0.3, with Biocondcutor version 3.12. For running SV callers, it is recommended to use a HPC environment on Linux.

**Sniphles**: The Sniphles workflow ( Figure 5) requires the following dependencies: Python >= 3.6, Pysam (Version 0.16.0) ( https://github.com/pysam-developers/pysam), Cyvcf2 (Version 0.30.2), ^60^ Sniffles (Version 1.12), ^10, 39^ SURVIVOR (Version 1.0.7), ^59^ Mosdepth (Version **0.2.6**), ^61^ Bcftools (Version 1.9) ^65^, tabix (Version 1.8). ^62^ The workflow partitions reads in the bam file into groups based on phase blocks and phase status, which enables parallel analysis of the data. The read coverage at each block and each phase is computed with Mosdepth and used to estimate the parameters for calling SVs by Sniffles. Next, the identified SVs per haplotype are concatenated using bcftools. SVs of two haplotypes are combined using SURVIVOR with option “1000 1 0 0 0 0” to merge SVs within 1 kbp between each other and to allow for different types of variants to be considered on different haplotypes. SVs are then force called with Sniffles using this combined vcf file. Force called SVs from each haplotype are combined with SVs of unphased regions as the final output ( Figure 5). To facilitate workflow testing, Princess ( https://github.com/MeHelmy/princess) was used to align, detect and phase SNVs and SVs from PacBio HiFi reads. The produced Bam from the previous step is the input for Sniphles, where pysam was used for alignment.

**Swagg:** The minimal system requirements for SWAGG are 8Gb RAM, 1 CPU and 10Gb of storage. Figure 6 demonstrates the implementation and operation of the SWAGG software package. Protein graphs are generated using a multiple sequence alignment of the proteins, then using the tool msa_to_gfa ( https://github.com/fawaz-dabbaghieh/msa_to_gfa) after which this multiple sequence alignment is converted into a graph file in GFA format, with the original sequences embedded as paths in the graph for visualization. This tool is tested with Python 3 and does not require any extra libraries or dependencies. New sequences can be aligned to these graphs using Partial Order Alignment algorithm for example.

**PanOriginSV:** PanOriginSV has three additional open source dependencies which are MMSEQ2 (for clustering), BCALM (pangenome creation) and GraphAligner (for sequence-to-graph alignment). MMSEQ2 is the most memory intensive step, and MMSEQ2 requires roughly 1 byte per sequence residue. An overview of the pipeline utilised by this tool is highlighted in Figure 7. PanOriginSV performs lab-of-origin prediction in three distinct steps. Firstly, during the training phase PanOriginSV clusters similar sequences using MMSEQ2. Further, the most similar clusters having a predefined number of representative lab labels are selected for the pangenome creation. Second, in order to incorporate SV information into the pangenome a graphical pangenome is created using BCALM for each of the clusters identified in the first step. These pangenomes reflect sequence-level structural variation that reveal important differences in highly similar sequences that could belong to different labs thereby reducing the possibility of false positives. The training sequences are then mapped back to their corresponding pangenome graph to obtain important alignment information including but not limited to number of hits and percentage identity of alignment. These are then collated and embedded into a feature vector that is passed on to a machine learning model for prediction.

Thirdly, features from the alignment steps are combined with sequence metadata and input into a random forest classifier that is trained to predict lab-of-origin in a multiclass classification task. Both the top-1 and top-10 predictions are output and compared to previous literature and Genetic Engineering Attribution Challenge (GEAC) benchmarks.

**GeneVar**: Figure 8 shows the different components of GeneVar, which is a web browser application. The webpage, including data storage, requires only one core with 1 Gb RAM and requires less than 1 Gb of storage. After entering the gene name (HGNC, Ensembl gene (ENSG), or transcript (ENST) identifier) in the search box on the homepage, you will be directed to the gene-specific page containing: 1) Gene-level summary with number of SVs, number of clinical SVs or SVs overlapping clinical SNVs, 2) Links to the gene's page on OMIM, GTEx, gnomAD, 3) A dynamic table with the annotated variants overlapping the gene, 4) A graph with the distribution of the allele frequency for variants matched with gnomAD-SV (50% reciprocal overlap). The profile of the SV to consider, such as type and size range, can be specified on the side bar. Each column in the dynamic table can be "searched" into or reordered dynamically. All data used by the app will be available for download in tab-delimited files. By default, allele frequency is reported based on dbVar ^63^ and gnomAD genomes and exomes. Furthermore, GeneVar utilises dbVar database and links SV to genes and annotate gene impact, allele frequency, and the overlap with clinically-relevant SVs, SNVs and indels. All data, are available for download in a tab-delimited file. Each variant has been extensively annotated and aggregated in a customizable table. GeneVar is available on GitHub ( https://github.com/collaborativebioinformatics/GeneVar). The repository provides detailed instructions for tool usage.

**SVTeaser:** SVTeaser generates regions from a user provided reference and adds in a structural variant into each region using one of two methods - 1) a call to SURVIVOR simSV ^59^ which generates random, simulated SVs by introducing variation (deletions (DEL) and insertions (INS) type of SV breakpoints) in DNA sequences, or 2) automatic spike-in of a known SV from an input SV VCF file. Resultant altered reference sequences are then used for Illumina short-read simulation using ART. ^64^ Parameters controlling simulated sequencing read-length, insert-size, and depth parameters can be altered. Simulated reads can then be mapped to the original, unaltered reference with any mapper of choice; here, BWA was used. Resultant BAM files can then be used to detect SVs using any mapping-based SV caller of choice; here, Parliament2 ^40^ was used to generate calls with manta, breakseq, ^65^ cnvnator, and lumpy. The resultant VCFs are then matched to the simulated SVs’ VCFs using Truvari and output is parsed into a pandas dataframe for report generation. SVTeaser requires installation of Python 3.7, Truvari, SURVIVOR, ^59^ Vcftools and ART read simulator. The components of SVTeaser are shown in Figure 9.

**XSVLen:** The XSVLen workflow requires Python3, Minimap2, Nextflow, and R >=3.5.0. As demonstrated in Figure 10, XSVLen takes as input a haplotype-resolved *de novo* assembly, and a VCF file (generated by cuteSV ^39^ or sniffles ^10^) of variants including only insertion and deletion calls. For each insertion or deletion call within the vcf file, a modified reference genome is generated. This modified reference will contain a 1.5kb flanking sequence that either has the sequence removed if a deletion call, or the alternate sequence added between the flanking sequences if an insertion call. The resulting ‘query’ sequences are then mapped using minimap2 to both haplotypes. Each aligned query gives rise to a map of aligned bases P={(q _1_,t _1_), … , (q _n_,t _n_)}. To score each variant call, we find two indexes i, j. These index the end of the prefix, and beginning of the suffix in the query. When the call is valid, (P [j][0] - P [i][0]) - (P [j][ 1] - P [j][0]) is equal to 0. To account for differences in alignment, we iteratively search for an (i ^opt^, j ^opt^) combination, with i ^opt^ ≤ i and j ≤ j ^opt^ that gives the smallest difference. Variants are reported as valid if the difference is less than 10 bases or the intervals defined between P [i ^opt^] and P [j ^opt^] are within 95% length in either haplotype. A summary report is then produced using an R script.

### Use Cases

**NibbleSV:**

We benchmarked NibbleSV over the GIAB HG002 SV call set ^66^. In summary, this call set was created using multiple long and short read technologies and underwent manual validation across multiple groups to ensure an overall high quality and accuracy. Using an Illumina data set from (2x250 GIAB HG002) we benchmarked true positives (i.e. SV that should be present), false positives (i.e. parental only SV that should not be present in the proband HG002), and false negatives (i.e. SV that should be present in HG002 but were not found). Using only chr 22 from HG002, NibbleSV with a kmer size of 23 takes around 2-4 minutes on a single thread with a 80gb bam file and the provided VCF file. We assessed our recall at different k-mer sizes which increases with the kmer size. For example, k=21 (2min 3sec) achieves 0.59 recall with a precision of 0.83. Interestingly, for insertions the recall rate increases to 0.86 with a precision of 0.86.

**Figure 11.**
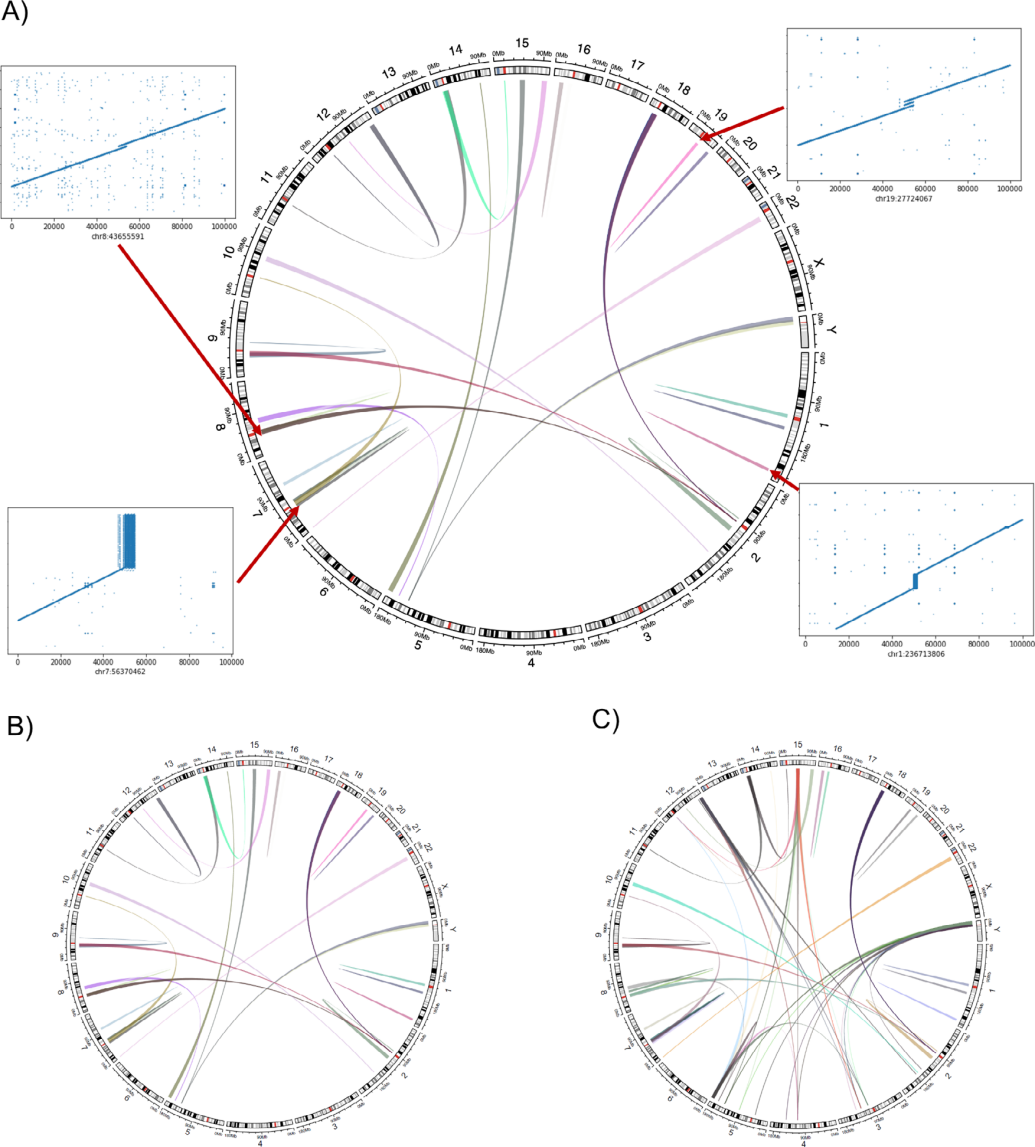
Relationship between the CNV calls and identified SVs across the CHM13 genome. CNV2SV results using CHM13 in comparison to GRCh38. (A) The diagram connects the genomic location of individual CNV calls on GRCh38 (broad ends) to the location of their linked SV identified in the GRCh38-CHM13 genome-genome alignment (thin ends). The diagram reveals a number of underlying putative translocation events identified for the called CNVs. Adjacent CNV-SV links (putative tandem duplications) are shown as streaks at the respective genomic position. For four select CNV-SV links, the genome-genome alignment for the underlying SV is further shown as a dot plot on the outside of the diagram. Only the best matching SV link (thin end) identified for each CNV call (broad end), as determined by sequence alignment score between both events, is shown here. (B) Similar to A, but displaying all potential CNV-SV links that meet the default alignment identity threshold of 0.8. CNVs with multiple matching duplication SV events identified in the genome-genome alignment can be explained by copy numbers greater than two occuring in distant locations, and alternatively may suggest the involvement of complex genomic rearrangements including transposable elements.

**Figure 12.**
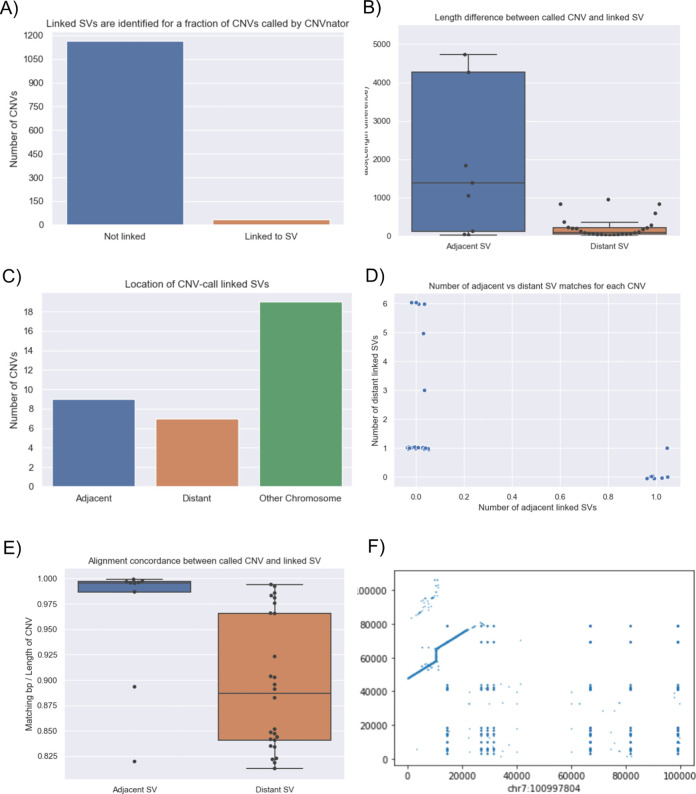
Summary the linkage statistics showing the characteristics of disparities between the CNV calls and SVs. In terms of linkage statistics, the majority of the CNVs identified have not been linked to a SV event, as indicated by **A**. One of the main reasons for the unsuccessful linking is the length disparity between the called CNV events and SV events as shown in **B**. Overall, among linked CNV and SV events, the distribution of three major categories are shown in **C**: adjacent events, distant events, and events spanning multiple chromosomes. Distant events are called in the case when the linked SV is at least 1Kbp away from the CNV call (either upstream or downstream). Details of the distribution of the adjacent and distant events per CNV call are given in **D** and **E** shows that alignment quality is better for adjacent matches when compared to more distant SV matches. While most links for a single CNV event are unanimously distant or adjacent, we observed an event in which a CNV was linked to both an adjacent and a distant SV which occurs on chromosome 7 ( **F**: adjacent: chr7:100997804 length 8325 and distant: chr7:100994092 length 3249).

**CNV2SV**:

Figure 11a shows the best links (based on the alignment identity score) between CNVs identified by CNVnator and duplication SVs inferred from the dipcall alignment of CHM13 and GRCh38. The genomic areas surrounding four selected adjacent duplication events are further highlighted using dot plots, revealing the architecture of the corresponding variation in the context of the genome-genome alignment. In Figure 11b, all CNV-SV links meeting a default alignment identity threshold are shown, further revealing events corresponding to putative copy number increases of greater than two. We further explored the reason why some CNV and SV could not be linked, through statistical analysis of the raw SV calls as shown in Figure 12.

**Figure 13.**
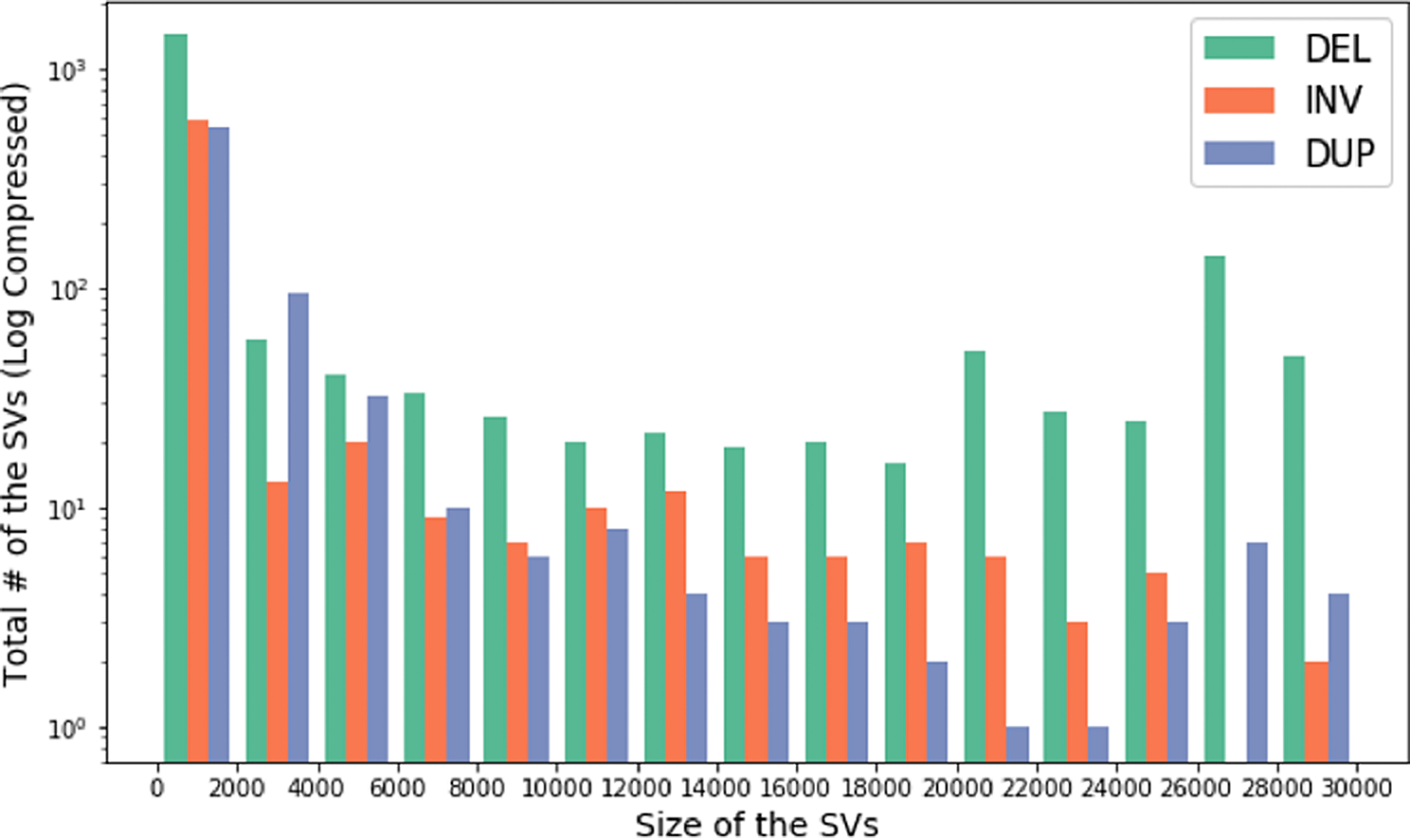
An analysis of size and number of identified SARS-CoV-2 SVs. Histogram showing size of the SVs and the total number of SVs across multiple Nanopore and Illumina datasets. The y-axis of the histogram is log compressed.

**CoronaSV**: We processed more than 200 SARS-CoV-2 SRA runs with CoronaSV, and Figure 13 shows the high confidence SVs generated with SURVIVOR ^59^ by taking a majority vote across multiple SV callers. We also looked for shared SVs across multiple samples. There were only a few inversions identified that were consistently and reliably called between samples. We believe those inversions are related to the transcriptional landscape of SARS-CoV-2. These inversions are small (less than 1Kb), and five of them were found in ORF1ab and one on ORF M.

**Figure 14.**
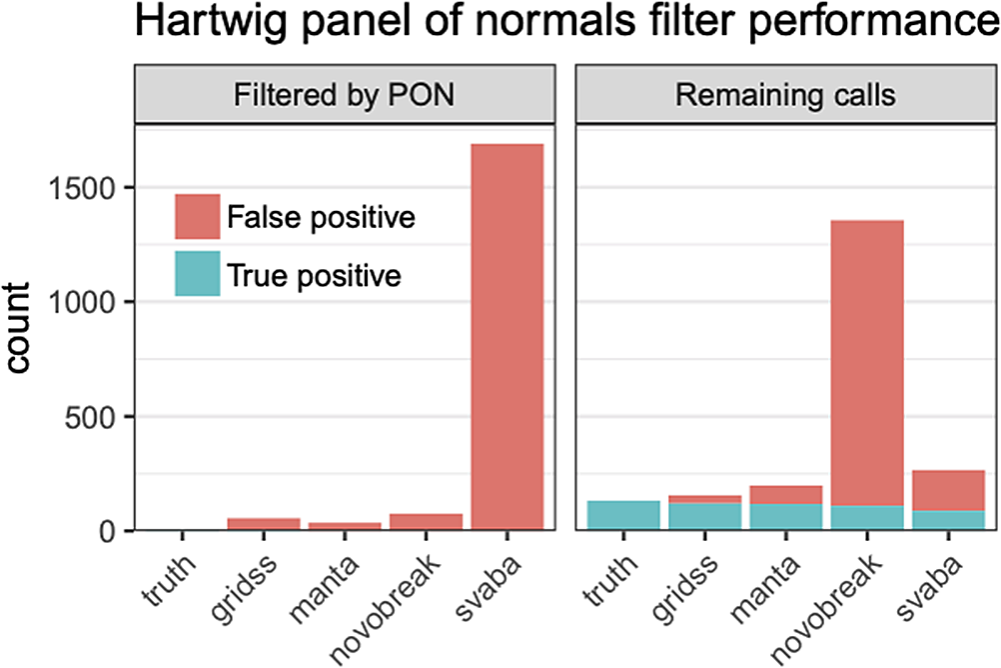
Filtering somatic SV calls using a Panel of Normals. Using a cohort of 3,792 freshly-sequenced WGS normal samples to create a Panel of Normals (PON) from GRIDSS calls and a set of curated calls from sample COLO829 to classify false positives, we discover that a sizeable number of false positives were found within the panel of normals, suggesting that these were miscategorized due to algorithmic errors. PON filtering based on GRIDSS calls is effective for the modern callers such as GRIDSS, Manta and SvABA which (partially) rely upon localized assembly.

**CleanSV:** We investigated filtering SV calls using both the curated reference set and a high-quality panel of normals (PON). The PON was created using GRIDSS calls from 3,782 normal samples freshly-sequenced at a median depth of 38x by the Hartwig Medical Foundation ^49, 50^. Using this PON consisting of GRIDSS calls from 3,792 freshly-sequenced normal WGS samples, we explored how the percentage of calls from short-read SV callers which were incorrectly labeled as true somatic calls, either due to being algorithmic artifacts or germline calls. Our results show the promise of such an approach. ( Figure 14). Note that this is not only a check for false positives: we know a priori that many calls from somatic SV callers are mislabeled as somatic when they’re actually germline. This is a known algorithmic error: SVs are normally first called in the normal sample and labeled as “germline”, and then the resulting SVs called using the tumor sample (separately or jointly with against the normal) are labeled as “somatic”. While such an approach is common for short-read SV callers, this frequently leads to mislabeled results, often for the simple reason that the normal sample is sequenced at a much lower coverage than the tumor sample.

We plan to continue to explore whether the false positives found exhibit distinct features which we could use for future filters to distribute to the community. With these insights, given that clinical genomics still overwhelmingly relies upon short-read sequencing, our goal would be to also apply filters to all variants (e.g. the case of Mendelian diseases).

**Sniphles**:We used Princess ( https://github.com/MeHelmy/princess) to align, detect and phase SNVs and SVs from PacBio HiFi reads 32x coverage. The produced Bam from the previous step is the input for Sniphles, where pysam ( https://github.com/pysam-developers/pysam) was used for alignment. For each phase block we used the mosdepth ^61^ to detect coverage. Later, we called SVs using Sniffles ^10, 39^ with the adequate numbers of reads to support SV. The identified SVs per phase block were sorted and concatenated using bcftools version 1.9 ^67^, and both the haplotypes were merged using SURVIVOR.

**Figure 15.**
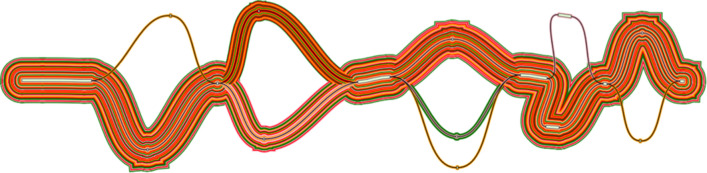
SARS-COV-2 nucleocapsid protein graph. Protein graph with paths representing the original sample. This graph here is a directed acyclic graph of an MSA of 26 “N” gene (Nucleocapsid Phosphoprotein) generated from 26 SARS-COV-2. Visualized using gfaviz. ^70^

**Swagg:** The main results from the Swagg package includes the development of the graph module and the protein graphical application ( Figure 15). The graph module is able to retrieve basic statistics from a pangenome graph in GFA format from either reference-based (fasta + VCF) or a set of assemblies (fasta), followed by conducting a pairwise comparison of all paths in the graph, and outputting a matrix in TSV format with the path names and corresponding samples in the first position. After creating the pairwise matrix, the module can plot an SV pileup over any path in the graph, counting the number of other paths that contain an SV overlapping each position. In addition to utilizing the pairwise comparisons where hotspots are references to a given sample, this approach also allows for graphs derived from vcf files. The objective from the protein graph mapping application was to show if the variants introduce new amino acids or stop a stop codon. Similarly, a pangenome graph can be constructed from DNA sequences as panproteome graphs can be built from different amino acid sequences of a protein. These graphs can then help visualize the variants between the sequences and show the paths each sequence take through the graph. Another layer of information can be added to the nodes, e.g. does a node represent a conserved or a non-conserved side, does a path divergence in the graph has any significant phenotypic characteristics, relating genome-wide association studies to these proteins graph ^68^. Therefore, when aligning a new sequence to the graph, one can check the path the new sequence took in the graph and what information are related to this path. ^69^ Figure 15 shows an example of an annotated graph of the Nucleocapsid Phosphoprotein in SARS-COV-2.

**PanOriginSV:** It became apparent that the quality and representation of the clusters was a main factor in prediction accuracy. To this end, we tested PanOriginSV on a range of clusters of at least 500 sequences and only considered cases where the test sequence had a training representative in the assigned cluster. The CPU time used by PanOriginSV was 10-50x less than the linear model (depending on the cluster). We observed a range of results, with the linear prediction model outperforming PanOriginSV by up to 5% in some clusters and PanOriginSV outperforming the linear model by up to 6% in others ( Table 1). With deeper analysis of the input data, we hope to achieve better clusters and thus better prediction results with the graph model. It is also worth noting that our graph construction method can be improved using more recent pangenome graph construction tools.

**Table 1.** Summary of PanOriginSV prediction accuracy. Benchmarking results on large clusters obtained from MMSEQ2. For a single cluster, 25% of sequences were held out and used as the testing set. The accuracy of the top predicted lab was consistently higher in PanOriginSV compared to PlasmidHawk, however the accuracy when testing against the top 5 predictions for both tools was comparable.

Cluster	Train Sequences	Test Sequences	Test accuracy (Linear)	Top 5 test accuracy (Linear)	Test accuracy (Graph)	Top 5 test accuracy (Graph)
J7OEM	2870	714	0.96	0.99	0.97	0.99
3PTDM	2397	571	0.82	0.93	0.81	0.93
O3GQU	1046	275	0.65	0.88	0.71	0.83
48073	973	205	0.71	0.89	0.71	0.87
WA905	639	149	0.87	0.94	0.87	0.97
GIGX0	604	119	0.71	0.87	0.79	0.93

**Figure 16.**
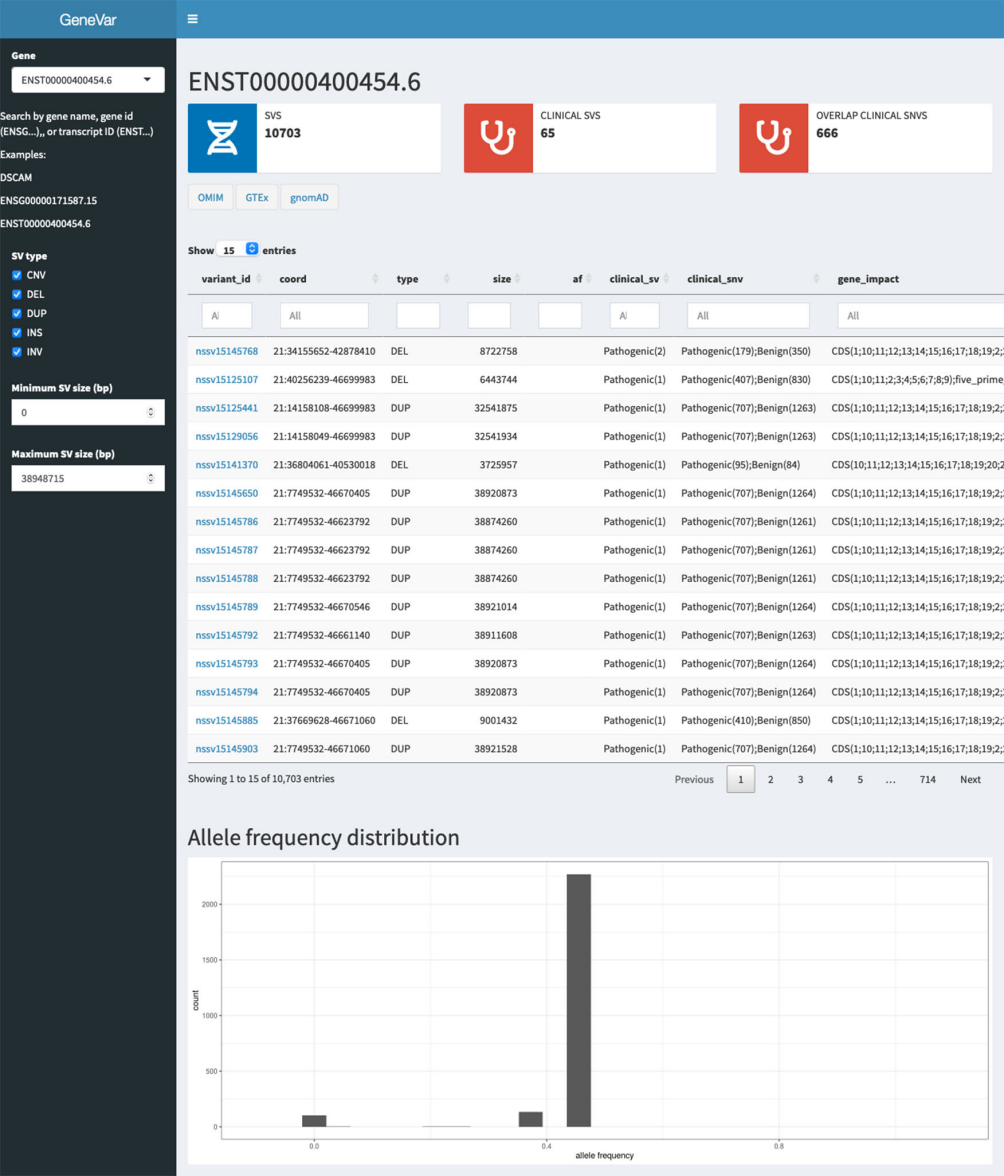
User interface of GeneVar data browser. A description of the elementary transcript details for the gene of interest. This includes the Ensembl transcript ID, Ensembl Gene ID, number of exons and genomic coordinates as described in the GRCh38 build.

**GeneVar**: **Databrowser.** Upon querying a gene or transcript, the data browser will visualize a rare-variant burden test, allele frequency distribution, and variant level information for known SVs within dbVar. The data browser does not have a login requirement and integrates multiple public resources ( Figure 16). To illustrate whether a particular gene/transcript or exon has been adequately covered to detect variation, the average depth of coverage is graphically represented. An additional panel shows gene expression levels across all general tissues included in GTEx ^71^. **Report summary**. Analysis results are enriched with information from several widely used databases such as ClinVar ^72^ and gnomAD, ^73^ as well as graphical visualization utilities integrated in the pipeline as part of GeneVar. Resulting variants are reported in a tab delimited format to favor practical use of worksheet software such as iWork Number, Microsoft Excel or Google Spreadsheets. All information can be downloaded in tabular form.

**Figure 17.**
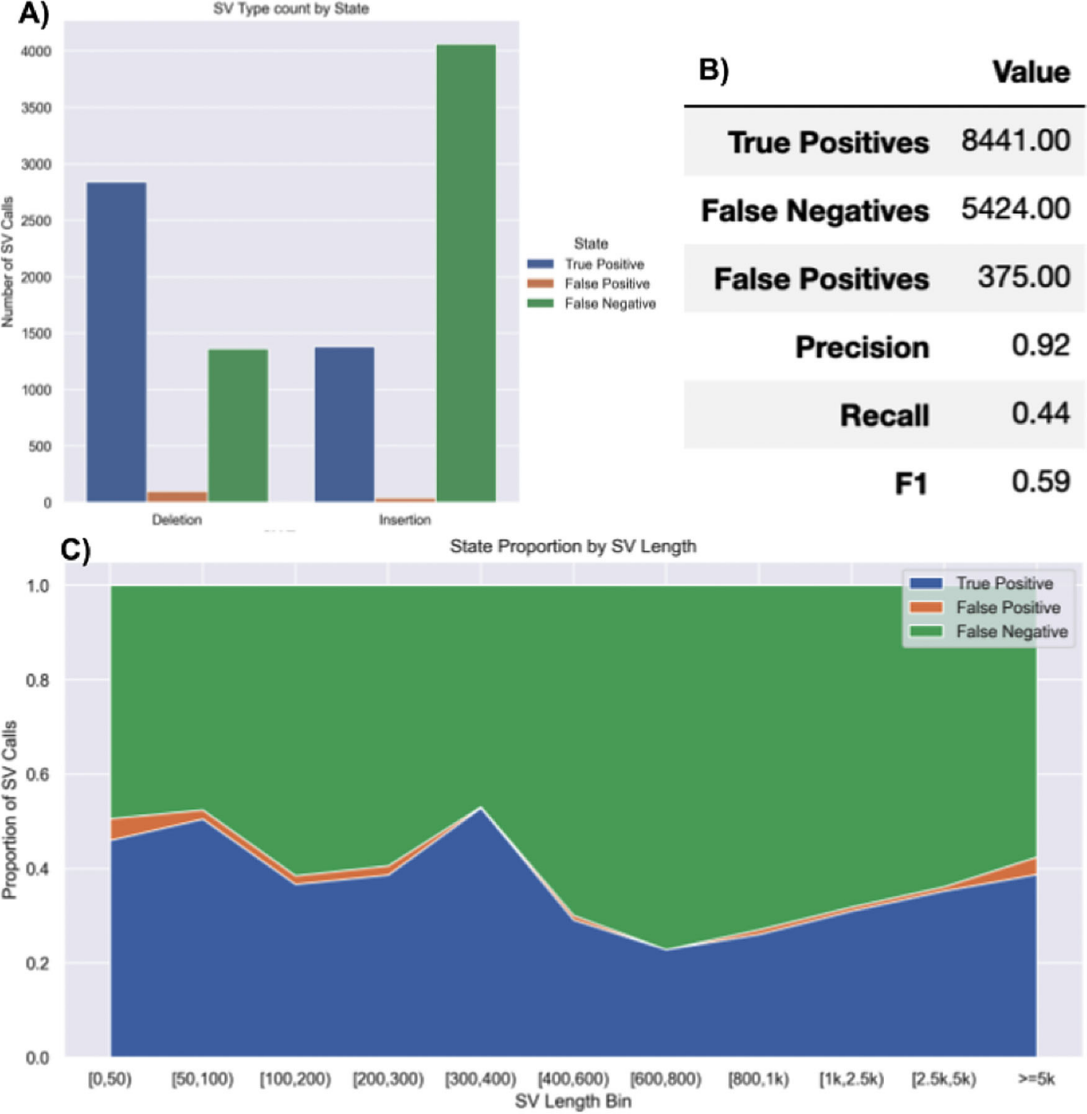
Summary report output of SVTeaser. A report generated from benchmarking a real HG002 Manta 30x Illumina sample against GIAB Tier1 SVs. A) Counts of SVs by SVType and their intersection state with GIAB Tier1 benchmark SVs. B) Summary table of benchmarking performance. C) Proportions of SV intersection states with the benchmark by SV size bin.

**Figure 18.**
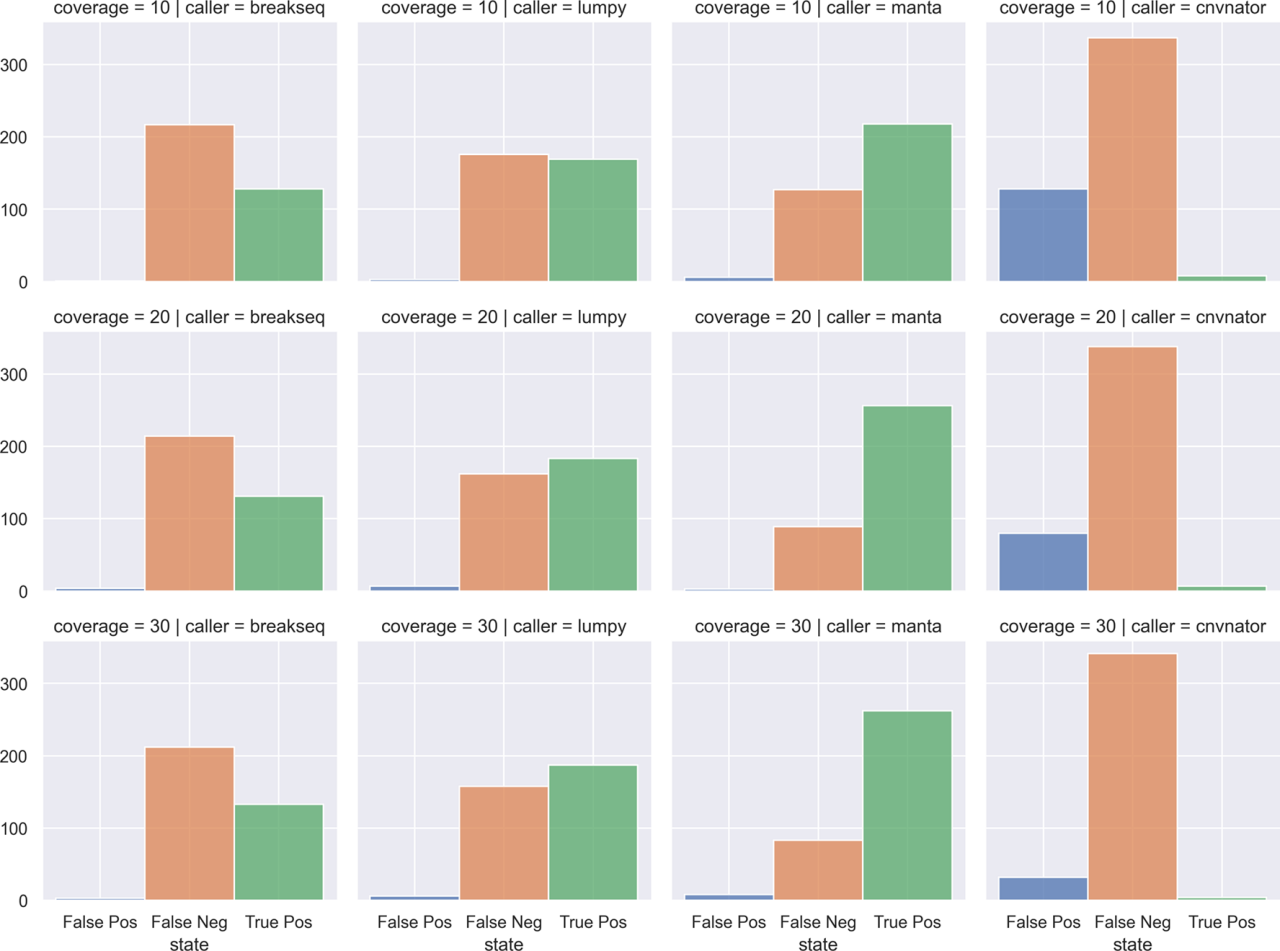
A comparison of simulated DELs across SV callers and sequencing coverages. Count of False Positives, False Negatives, and True Positives from four SV callers (columns) against chromosome 2 deletions through multiple simulated coverages (rows). SV callers: breakseq, lumpy, manta, cnvnator. Coverages: 10x, 20x, 30x.

**SVTeaser**: SVTeaser was able to simulate SV data on average 14 min per sample when tested on chromosome two. SVTeaser output includes organized VCF results of true positive, false positive, and false negative from evaluated SV callers. Furthermore, performance scores are reported alongside automated plots for quick visual evaluation of SV callers ( Figure 17). SVTeaser is able to simulate sequencing for known deletions and insertions with various coverage, read length, and insert length. This enabled thorough evaluation for understanding the strengths and boundaries of the SV callers in question ( Figure 18).

**XSVLen**: A diploid assembly of the MHC locus of HG002 from Nurk *et al*^74^ was used to benchmark variants. The assembly has an N50 of 16.1 and 18.0 Mb per haplotype. Variants were called using 50-fold coverage of ONT reads using CuteSV version v1.0.8. The number of INS and DEL per SV size can be observed in Table 2. The number of INS/DEL overlapping with the haplotype-resolved assemblies are shown in Table 2 as well as a comparison of assembly-based calls and gold-standard Truvari calls.

**Table 2.** Summary: Insertion and deletion events are compared according to structural variant size and number and overlaps with 1) haplotyped assemblies and 2) Truvari callsets are counted.

SVType	Insertions		Deletions	
SVLen	<50	>=50	<50	>=50
Number of insertion/deletion calls by cuteSV	20007	14942	38331	10926
Number of these calls that overlap assembly contigs	16054	17481	30494	6475
Number of truvari TP/FP/no-call (NP)	3351 FP 7200 TP 15974 NP	2835 FP 4095 TP 80 NP	880 FP 1925 TP 30196 NP	1571 FP 2099 TP 298 NP
Number of assembly TP/FP/no-call (NP)	5220 FP 10834 TP	8979 FP 8502 TP	23202 FP 7292 TP	3444 FP 3031 TP

## Conclusion

The results of the 2020 Baylor College of Medicine/DNANexus hackathon described here represent novel work that pushes the field forward for human genome SV detection but also for Covid related research. Both are needed to further current findings about diversity and the complexity of organisms and their genotypes. To further facilitate this progress in a FAIR-compliant manner, 80 people came together from across the world in October 2020 completed 10 groundbreaking prototypes. Hackathons like these not only represent short bursts of prototype development, but are essential to form groups and communities, inspire communication across countries and research institutions, and form novel collaboration networks. As such, this year’s hackathon not only sparked the projects described here, but also highlighted the need for unified databases for SVs and other genomic features hosted on DNAnexus, Anvil, and other platforms as well as larger standards and references (e.g. GIAB, UKBB). This is essential to ensure quality standards for benchmarking and comparability, which will further advance the science and medical research.

Rapidly switching to a completely remote hackathon that enabled increased international participation was made necessary by the COVID-19 pandemic. This allowed for an open science effort across an even more diverse population of individuals and professional backgrounds. That diversity made it possible to complete 10 projects, which spearheaded novel insights in the understanding of structural variants in humans, as well as COVID19 genome structure. More importantly, it led to new synergies among participants, an active online community, and new friendships across borders.

## Data availability

**Associated code is available at:** DOI 10.17605/OSF.IO/ME62X

**Data sources utilized:**

NibbleSV: Genome in a Bottle, HG002, SV callset ( ftp://ftp-trace.ncbi.nlm.nih.gov/ReferenceSamples/giab/data/AshkenazimTrio/HG002_NA24385_son/PacBio_CCS_15kb/)

CNV2SV: CHM13 ( https://github.com/nanopore-wgs-consortium/CHM13#telomere-to-telomere-consortium) and GRCh38

CoronaSV: Data sources available on https://github.com/collaborativebioinformatics/coronasv

CleanSV: hs37d5 HG002 BAM ( ftp://ftp.1000genomes.ebi.ac.uk/vol1/ftp/technical/reference/phase2_reference_assembly_sequence/hs37d5.fa.gz), hg19 COLO829 BAM (https://nextcloud.hartwigmedicalfoundation.nl/s/LTiKTd8XxBqwaiC?path=%2FHMFTools-Resources%2FGRIDSS-Purple-Linx-Docke r)

SWAGG: Data sources available on https://github.com/collaborativebioinformatics/swagg/blob/main/sample_manifest.tsv

XSVLen: MHC locus of HG002 (74)

GeneVar: Data sources available on https://github.com/collaborativebioinformatics/GeneVar

PanOriginSV: genetic engineering attribution challenge (GEAC)

https://www.drivendata.org/competitions/63/genetic-engineering-attribution/

SVTeaser: All data utilized was based on simulations

Sniphles: Genome in a Bottle, HG002, SV callset ( ftp://ftp-trace.ncbi.nlm.nih.gov/ReferenceSamples/giab/data/AshkenazimTrio/HG002_NA24385_son/PacBio_CCS_15kb/)

## Software availability

**NibbleSV**

Source code available from: https://github.com/collaborativebioinformatics/nibSV

Archived source code at time of publication:

License: MIT license

**CNVSV**

Source code available from: https://github.com/collaborativebioinformatics/CNV2SV

Archived source code at time of publication:

License: MIT license

**CoronaSV**

Source code available from: https://github.com/collaborativebioinformatics/coronasv

Archived source code at time of publication:

License: MIT license

**CleanSV**

Source code available from: https://github.com/collaborativebioinformatics/CleanSV

Archived source code at time of publication:

License: MIT license

**Sniphles**

Source code available from: https://github.com/collaborativebioinformatics/Sniphles

Archived source code at time of publication:

License: MIT license

**Swagg**

Source code available from: https://github.com/collaborativebioinformatics/swagg

Archived source code at time of publication:

License: MIT license

**PanOriginSV**

Source code available from: https://github.com/collaborativebioinformatics/PanOriginSV

Archived source code at time of publication:

License: MIT license

**GeneVar**

Source code available from: https://github.com/collaborativebioinformatics/GeneVar

Archived source code at time of publication:

License: MIT license

**SVTeaser**

Source code available from: https://github.com/collaborativebioinformatics/SVTeaser

Archived source code at time of publication:

License: MIT license

**XVSLen**

Source code available from: https://github.com/collaborativebioinformatics/The_X_team

Archived source code at time of publication:

License: MIT license
