# Supplementary material for: An international virtual hackathon to build tools for the analysis of structural variants within species ranging from coronaviruses to vertebrates
Source: F1000Res. 2021 Sep 3;10:246. Originally published 2021 Mar 26. [Version 2] doi: 10.12688/f1000research.51477.2 (PMC8479851; doi:10.12688/f1000research.51477.2)
Supplement: Peer review history

# Peer review: Reviewer response for version 2

**DOI:** 10.5256/f1000research.76830.r93372

**Published:** 2021-09-28

**Authors:** Francois Sabot

**Affiliations:** 1 DIADE, Univ Montpellier, IRD, CIRAD, Montpellier, France

The current version of the McCartney paper on the virtual hackathon is ok for me. I just saw a few formatting errors.

Are the conclusions about the tool and its performance adequately supported by the findings presented in the article?

Partly

Is the rationale for developing the new software tool clearly explained?

Yes

Is the description of the software tool technically sound?

Partly

Are sufficient details of the code, methods and analysis (if applicable) provided to allow replication of the software development and its use by others?

Partly

Is sufficient information provided to allow interpretation of the expected output datasets and any results generated using the tool?

Partly

Reviewer Expertise:

Genomics, bioinformatics, evolution

I confirm that I have read this submission and believe that I have an appropriate level of expertise to confirm that it is of an acceptable scientific standard.

# Peer review: Reviewer response for version 1

**DOI:** 10.5256/f1000research.54651.r82807

**Published:** 2021-06-07

**Authors:** Loren L. Flynn

**Affiliations:** 1 Murdoch University, Murdoch, WA, Australia

McCartney and colleagues describe a compelling and comprehensive account of the outcomes of an international hackathon to develop new tools for the discovery and analysis of structural variants (SVs). The authors describe 10 novel software tools to overcome gaps in the available tools to more accurately identify, genotype and map SVs, as well as provide predictive functional effects of the variants to gene expression. The identification of new and accurate tools to identify SVs is of critical importance for understanding the genetic underpinnings of complex disease with no monogenic pattern of disease inheritance. This manuscript is the product of a virtual international hackathon, describing newly developed tools as a foundation for future discussion and development, and the links to the already available online tools provide additional and necessary information for understanding the software applications and outputs. I recommend this manuscript for indexing following amendments. From a functional genetics perspective, I provide the following comments and suggestions:

 1. The manuscript does not provide full detail about the code, methods and analysis of the individual tools, however, the purpose of the manuscript is to describe the hackathon and the resultant projects, with the intention for future development of these tools. This could be made clearer in the text with the development status and future requirements for each tool discussed.

 2. A summary table of the main applications, benefits, and stage of development for each of the tools and the future direction would be helpful for readers. In particular, more information on the specific applications of each of the tools would be helpful for those reading the manuscript without a bioinformatics background.

 3. The manuscript is written with an introduction to each tool, the operation for each and finishing with use cases or results for each. As a suggestion, describing each tool separately under its own heading and incorporating each of these sections would improve the flow of the manuscript.

 4. The current tools for accurate SV calling and genotyping from GWAS data are limited for poly-nucleotide variations. Do any of the described tools address this limitation? 

 5. The Swagg software describes a tool for SV calling and mapping at both the gene and protein level. However, the advantage of SV calling and mapping at the protein level is not clear given that many SVs are found in non-coding regions. The protein function is not further described in the methods and the interpretation of the figure in the results section was not clear. It would be useful to provide more information for the protein mapping. Can this software show predictive changes to mRNA or protein structures induced by SVs in non-coding regions?

 6. The GenVar tool offers a clinically relevant analysis with predictions to SV effects on regulation of gene expression. Does the tool identify SV regions based only on the reference sequence or is it able to further compare a patient sequence to the RefSeq and provide information as to the carriage of potential risk variants/variants known to effect gene expression?

 7. There is no figure displaying the result output for the phase sequencing analysis Sniphles tool, this would help in explaining the data analysis and output.

Are the conclusions about the tool and its performance adequately supported by the findings presented in the article?

Partly

Is the rationale for developing the new software tool clearly explained?

Yes

Is the description of the software tool technically sound?

Partly

Are sufficient details of the code, methods and analysis (if applicable) provided to allow replication of the software development and its use by others?

Partly

Is sufficient information provided to allow interpretation of the expected output datasets and any results generated using the tool?

Partly

Reviewer Expertise:

Genetic therapies, functional genetics

I confirm that I have read this submission and believe that I have an appropriate level of expertise to confirm that it is of an acceptable scientific standard, however I have significant reservations, as outlined above.

# Peer review: Reviewer response for version 1

**DOI:** 10.5256/f1000research.54651.r83790

**Published:** 2021-05-20

**Authors:** Ricardo Assunção Vialle

**Affiliations:** 1 Nash Family Department of Neuroscience & Friedman Brain Institute, Icahn School of Medicine at Mount Sinai, New York, NY, USA

This manuscript describes ten Structural Variation related software tools developed at the Second Baylor College of Medicine & DNAnexus hackathon. These tools successfully cover relevant topics in the SV field and certainly will be appreciated by the scientific community. As other reviewers pointed out, my primary concerns are that the text still needs some work to improve readability and fix inconsistencies (e.g., American vs. British spelling). Also, a table summarizing all tools described will be very welcome. There are also some issues specific to some tools that should be addressed. Comments for each tool are described below.

**NibbleSV**: The tool seems great, performs efficiently, and the results are impressive. I just have few comments that would be interesting to see. First, the authors could add more details about the tests using the HG002. If I understood right, the GiaB tier 1 v0.6 was used as candidates to genotyping using the Illumina reads (250bp), is that correct? Also, how was the evaluation done? Second, if possible, it would be interesting to see how NibbleSV compares with other tools (e.g., paragraph). Finally, it would be nice to see how it performs in more complicated regions (e.g., segmental duplications). I wonder if NibbleSV could help filter artifact SVs found by misalignment in those regions.

**CNV2SV**: The motivation and the tool description must be improved in the manuscript. Also, the results are not well described and discussed, being restricted to the legends of Figures 11 and 12.

*Minor:*

 In the methods, CNV2SV circularlize should be circlize to refer to the R package.

 Missing description of letter C in figure 11 legends.

**CoronaSV**: The method proposes a Snakemake pipeline for SV discovery in SARS-CoV-2 genomes. It integrates short and long reads SV discovery tools, as well as assembly-based methods. CoronaSV also handles downloading sequencing data from SRA for variant calling. As a use case, the pipeline was applied to over 200 genomes, and a basic report of the results was shown. I’m not familiar with viral genomes, but I’m wondering what is the actual gain of applying such complex pipeline instead of performing *de novo* assembly and getting the SVs from it instead.

**CleanSV**: The tool proposes a systematic way for filtering false-positive somatic SVs in cancer genomics. The approach uses germline SVs identified in normal tissue samples to find the best filtering thresholds for specific SV callers. There is no description of how to run the tool, and an example dataset could be added to the GitHub page.

**Sniphles**: More details could be added to the diagram of the pipeline, the calling and merging steps are oversimplified. Is there a reason why Sniffles need Mosdepth to estimate its parameters? It would be great to see how it compares with the “first” approach of phasing mentioned by the authors, using tagged reads to phase SVs.

**Swagg**: The motivation and methods are not very clear in the manuscript. It seems to me that the protein-graph is basically capturing small-variants. Where SVs fit in this context? The example showed in Figure 15 needs a more detailed explanation (e.g., what the color mean? Is there any SV affecting this protein?). Also, some phrases are repeated in the introduction and methods sections.

**PanOriginSV**: I’m unfamiliar with the topic addressed by this tool. For the little information I could get, the proposal seems very relevant, and the results look promising. In the introduction, the authors discuss the drawbacks of using machine learning approaches and highlight PlasmidHawk advantages, but in the end, propose another machine learning method? What is different in this method compared to existing ones? I’m wondering if extending that tool to work non-linear pangenomes would be possible. Also, it would be nice to see the same benchmarking against PlasmidHawk.

**GeneVar**: The proposal of GeneVar seems interesting and novel. However, not all features described in the manuscript seemed to be implemented. For instance, the authors state that a rare-variant burden test is performed but, I could not find that on the demo website. Also, the annotations are still very basic, the presentation of the overlap is not user-friendly. I would suggest improving how information is shown, with more summarized information about the overlap.

**SVTeaser**: SVTeaser is a framework for benchmarking SVs using simulations. Simulations are performed using SURVIVOR. Overall, the method seems well developed, and the contribution is relevant for the field.

**XSVLen**: The methods sound truly relevant and novel. I’m very interested in applying this method in the future. I’m wondering if a more refined report could be implemented. It would be interesting to see results summarized for each haplotype separately. In the manuscript, the results table is hard to understand. For example, the first row shows 14942 INS >=50, while the second row shows a higher number when I would expect just a subset based on the description.

Are the conclusions about the tool and its performance adequately supported by the findings presented in the article?

Partly

Is the rationale for developing the new software tool clearly explained?

Yes

Is the description of the software tool technically sound?

Partly

Are sufficient details of the code, methods and analysis (if applicable) provided to allow replication of the software development and its use by others?

Partly

Is sufficient information provided to allow interpretation of the expected output datasets and any results generated using the tool?

Partly

Reviewer Expertise:

Bioinformatics, genomics, genetics

I confirm that I have read this submission and believe that I have an appropriate level of expertise to confirm that it is of an acceptable scientific standard, however I have significant reservations, as outlined above.

# Response

**Published:** 2021-06-14

**Authors:** Ann Mc Cartney

**Affiliations:** NIH, USA

***We would like to thank Ricardo for their time on giving the authors of this manuscript feedback and comments. Upon receiving these comments the corresponding authors reached out to the developers of each of the tools developed as part of this hackathon, each of which have done their utmost to respond to each comment or question.***

****

**Comment 1: **General: This manuscript describes ten Structural Variation related software tools developed at the Second Baylor College of Medicine & DNAnexus hackathon. These tools successfully cover relevant topics in the SV field and certainly will be appreciated by the scientific community. As other reviewers pointed out, my primary concerns are that the text still needs some work to improve readability and fix inconsistencies (e.g., American vs. British spelling). Also, a table summarizing all tools described will be very welcome. There are also some issues specific to some tools that should be addressed.

***Response: We agree that the format of the manuscript is somewhat cumbersome to read, however this is a pre-requisite determined by the journal for manuscripts associated with hackathons.***

****

**Comment 2:** For the Developer of NibbleSV: The tool seems great, performs efficiently, and the results are impressive. I just have a few comments that would be interesting to see. First, the authors could add more details about the tests using the HG002. If I understood correctly, the GiaB tier 1 v0.6 was used as a candidate for genotyping using the Illumina reads (250bp), is that correct? Also, how was the evaluation done? Second, if possible, it would be interesting to see how NibbleSV compares with other tools (e.g., paragraph). Finally, it would be nice to see how it performs in more complicated regions (e.g., segmental duplications). I wonder if NibbleSV could help filter artifact SVs found by misalignment in those regions.

***Response: Yes, the tests were done using GIAB Tier 1 v0.6 truth SV set and alignment file of illumina 2x250 bp dataset for HG002 sample. The true positive count is the number of genotyped SVs that are not parental SVs i.e. HG2count > 0 and the FP count is the parental SVs i.e. HG2count=0. The parental exclusive SVs that are not genotyped are counted as TNs and the HG002 SVs that are not genotyped by nibSV are counted as FN. The following metrics are used to evaluate performance of nibSV:***

***Recall = TP/ (TP+FN); Precision = TP/(TP+FP); Accuracy = (TP+TN)/(TP+FP+FN+TN); False discovery rate = FP/(TP+FP). The performance of nibSV was compared with SVTyper. SVTyper can genotype only non-insertion (ie. here deletions) type SVs, so we compared nibSV with SVtyper only for deletion type of SVs. nibSv was run with parameters k=23 and m=0.***

***Tool,** **TP,** **FP,** **FN,** **TN,** **Recall,** **Precision,** **Accuracy,** **FDR***

***nibSV,** **12152,** **2055,** **17957,** **5248,** **0.4,** **0.86,** **0.47,** **0.14***

***SVTyper,** **5873,** **212,** **24236,** **7091,** **0.20,** **0.97,** **0.35,** **0.03***

***The high recall score of nibSV shows that it outperformed SVTyper for genotyping the non-insertion type SVs. The high precision rate and low false discovery rate of SVTyper is due to the very low number of total genotyped SVs. The Paragraph tools required to add the padding bases to the SVs in the truth set. We could not successfully run Paragraph without making any changes to the truth set. Therefore, we have not included that tool in this result.***

**Comment 3:** *For the CNV2SV Developers: The motivation and the tool description must be improved in the manuscript. Also, the results are not well described and discussed, being restricted to the legends of Figures 11 and 12. Minor:In the methods, CNV2SV circularlize should be circlize to refer to the R package.Missing description of letter C in figure 11 legends.*

***Response: Thank you for your assessment. We have restructured the respective sections in the manuscript in order to clarify the tool motivation and description of operation. We have also corrected the figure accordingly.***

****

**Comment 4: **For the CoronaSV Developers: The method proposes a Snakemake pipeline for SV discovery in SARS-CoV-2 genomes. It integrates short and long reads SV discovery tools, as well as assembly-based methods. CoronaSV also handles downloading sequencing data from SRA for variant calling. As a use case, the pipeline was applied to over 200 genomes, and a basic report of the results was shown. I’m not familiar with viral genomes, but I’m wondering what is the actual gain of applying such a complex pipeline instead of performing de novo assembly and getting the SVs from it instead.

***Response: The reviewer makes a good point with respect to de novo assembly from short reads, which would accurately capture small structural variants. However, given SARS-CoV-2 is sequenced from an intrahost population, which often is from metatranscriptomes (RNA-sequencing of a microbiome sample) containing subgenomic RNAs, SVs reflect the transcriptional landscape of SARS-CoV-2. Thus we feel it is of use to capture the concordance across short reads, long reads, and assembly for structural variants calling.***

****

**Comment 5: **For the CleanSV Developers: The tool proposes a systematic way for filtering false-positive somatic SVs in cancer genomics. The approach uses germline SVs identified in normal tissue samples to find the best filtering thresholds for specific SV callers. There is no description of how to run the tool, and an example dataset could be added to the GitHub page.

***Response: Thank you to the reviewer for the response. As mentioned in the previous reviews, our contribution to the hackathon was a proof of concept. Releasing the filters for general use would require more work specifically investigating tumors of a given sequencing depth and tumor content. The panel of normals used for the paper are accessible, as cited in the paper.***

****

**Comment 6: **For the Sniphles Developers: More details could be added to the diagram of the pipeline, the calling and merging steps are oversimplified. Is there a reason why Sniffles need Mosdepth to estimate its parameters? It would be great to see how it compares with the “first'' approach of phasing mentioned by the authors, using tagged reads to phase SVs **.**

***Response: We would like to thank the reviewer for the comments and questions. We agree with the reviewer regarding the merging step, while this is a prototype of the idea, we will be working on enhancing the merging step. Additionally, we stated that in the manuscript.***

****

**Comment 7: **For the Sniphles Developers: “Sniffles need Mosdepth to estimate its parameters''. Response: Sniffles use a minimum of 10 reads to call an SV, we suggest to use mosdopth to calculate the coverage for the region we call SVs in, so it could adapt to low and high coverage as well as technology used for sequencing (PacBio or Oxford Nanopore Technology).

 Comment 8: For the Sniphles Developers: ‘’It would be great to see how it compares with the “first'' approach of phasing mentioned by the authors, using tagged reads to phase SVs.”.

***Response: We will be working on that, as the second approach still needs more tuning.***

**Comment 9: **For the Swagg Developers: The motivation and methods are not very clear in the manuscript. It seems to me that the protein-graph is basically capturing small-variants. Where do SVs fit in this context? The example shown in Figure 15 needs a more detailed explanation (e.g What does the color mean? Is there any SV affecting this protein?). Also, some phrases are repeated in the introduction and methods sections.

***Response: We would like to thank the reviewer for their comments and questions regarding the manuscript. In response to their questions, the different colors are associated with different sequences used to build the graph, with each sequence taking a certain path in the graph, which yields the colors.***

****

**Comment 10: **For the PanOriginSV Developers: I’m unfamiliar with the topic addressed by this tool. For the little information I could get, the proposal seems very relevant, and the results look promising. In the introduction, the authors discuss the drawbacks of using machine learning approaches and highlight PlasmidHawk advantages, but in the end, propose another machine learning method? What is different in this method compared to existing ones? I’m wondering if extending that tool to work non-linear pangenomes would be possible. Also, it would be nice to see the same benchmarking against PlasmidHawk.

***Response: We’d first like to thank the reviewer for their feedback. While PanOriginSV does use machine learning, it has the advantage that random forests are much easier to inspect, train, and interpret than other ML models. In addition, the step of constructing the pangenome already constructs features of the sequence. The results of PlasmidHawk are shown as the “linear” model in Table 1 as opposed to PanOriginSV, the graph model.***

****

**Comment 11: **For the GeneVar Developers: The proposal of GeneVar seems interesting and novel. However, not all features described in the manuscript seemed to be implemented. For instance, the authors state that a rare-variant burden test is performed but, I could not find that on the demo website. Also, the annotations are still very basic, the presentation of the overlap is not user-friendly. I would suggest improving how information is shown, with more summarized information about the overlap. 

***Response: Thank you reviewer, for your thoughts and assessment. Indeed, GeneVar is a work in progress and what you see now is the result of only 3 days of work. The developers agree that to make this a public tool, additional time and effort is needed.***

****

**Comment 12: **For the SVTeaser Developers: SVTeaser is a framework for benchmarking SVs using simulations. Simulations are performed using SURVIVOR. Overall, the method seems well developed, and the contribution is relevant for the field.

***Response: We appreciate the reviewer's careful and detailed assessment of our work, as well as his complimentary remarks. We believe that our tool will be useful in genetic research on SV predictions.***

****

**Comment 13: **For the XSVLen Developers: The methods sound truly relevant and novel. I’m very interested in applying this method in the future. I’m wondering if a more refined report could be implemented. It would be interesting to see results summarized for each haplotype separately. In the manuscript, the results table is hard to understand. For example, the first row shows 14942 INS >=50, while the second row shows a higher number when I would expect just a subset based on the description.

***Response: Many thanks to the reviewer for pointing this out and for the interest in the method, we have updated the main table for including the right numbers and also to help comprehension of categories. This was due to a bug in the annotation script that has been fixed. We did not perform a detailed exploration of haplotype-overlapping calls or the generation of a more detailed report as this was a proof-of-concept of the method but we agree it would be interesting if future work is performed.***

***We would like to thank the reviewer for their kind comments and feedback and hope that they have addressed all questions or concerns posed.***

# Peer review: Reviewer response for version 1

**DOI:** 10.5256/f1000research.54651.r83792

**Published:** 2021-05-17

**Authors:** Kamil S. Jaron

**Affiliations:** 1 Institute of Evolutionary Biology, The University of Edinburgh, Edinburgh, UK

**General comments**

 This manuscript is a write-up report of an apparently very successful hackathon. Authors present an overview of 10 different approaches to solve various issues in the genomics world of structural variants, some of then sound really promising. I appreciated the modest tone authors haven chosen when describing the current state of each tools. All the tools have at least some exploratory value, and all of them are publicly available, therefore I have no major objections for the manuscript to be indexed.

 This being said, I do agree with the first reviewer, I would greatly appreciate if before diving into details of individual methods, a big picture overview would be provided, for example a table with short descriptions, urls and development stages would be really handy. Disclosing the development stages would be particularly useful for a reader to know what to expect if proceeding further in reading.

 I do think this manuscript will spark a lot of ideas in the SV community, so I also wondered, what are the contribution policies to individual tools? Many of the tools have some future plans and are not yet production ready. Who would be the people to contact if someone would be interested in contributing and helping out with finishing a tool that would be particularly helpful for their research project?

 The manuscript could benefit immensely from a big reorganisation. I do believe that classical Intro/Meth/Results organisation is not doing this manuscript a favour. It was really hard for me to keep track of ten threads in parallel and at some point I just gave up on top-bottom reading. I ended up scrolling back and forth between different sections to get information regarding one tool I tried to understand at the time. I also think most of the people will be mostly interested in one of the tools only when opening the manuscript for reading, therefore I would propose to keep the common Introduction and Conclusion sections but restructure the rest of the paper paper on per method basis.

 I also appreciated authors' effort to comply with FAIR principles. Although I have not check every tool, those I did were neatly documented. Thoughts regarding individual tools are listed bellow.

**NibblerSV**

 If I understand right. The genotyping using NibblerSV is done in two phases - 1. calculating a dictionary of specific kmers for each allele, I would call those diagnostic kmers (e.g. allele specific kmers absent in the reference genome) and 2. matching kmers of resequencing data to the diagnostic kmers. I did find the idea very clearly explained.

 The part I did not understand is how it's decided if a sample does or does not carry a variant. Does it need to have all the diagnostic kmers at a certain coverage? Or at least some proportion? What if the sample contains kmers of multiple diagnostic markers? Is it possible to genotype a heterozygous SV? Is there any measure of confidence in genotyped alleles? I personally would prefer to read more about these details over technical aspect such as different types of temporary files that the software is producing that actually are mentioned.

 I also wondered, given the approach of generating catalogues of SV-diagnostic kmers, I would anticipate very variable power to detect SVs of different sizes and types. For example, recent inversions will be identified only through very few kmers found at the edge of the inversion, compared to large scale insertions that will probably harbour many diagnostic kmers and clearer signal. Did you look at the sensitivity/precision in respect to size/type? Would that be a consideration one should have when deciding if to use nibSV or paragraph or VG?

**CNV2SV**

 This tool has the least intuitive motivation of all the tools presented. I would naturally assume all loci with CNV should also be called as SVs, hence I was not sure why it is interesting at all getting the same information twice. However, the results of this analysis proved my intuition wrong. Only very few CNVs are linked to SVs. However, the suggested explanation with length disparity was a bit dissatisfying to me. How comes that the SV and CNV callers call variants of so different size? Which are shorter/longer? Do you think the CNV/SV reconcilation could be more overlapping with a different choice of SV/CNV caller?

 This software has absolutely outstanding description on the GitHub repository.

*Minor comments:*

 Figure 12, panel B has a cropped y-axis label.

 In the Methods the text "formats (from Parliament" has an opening bracket that is never closed.

**CoronaSV**

 I did appreciate the idea of scalable pipeline for conservative analyses of SARS-CoV-2 genomes. Authors made many unjustified choices. For example, why did you chose in particular Manta, Delly, Lumpy and Tardis to detect SV using short reads? But I suppose it is understandable why it would be challenging justify every choice in limited time of a hackathon.

 The step of the analysis I am worried about is the merging by SURVIVOR, it also happens to be the only one that does not seem to be included in the GitHub repository. What parameters were used for individual merging steps using SURVIVOR? Given the genome of SARS-CoV-2 is really small, I believe that the outcome of this pipeline will be very sensitive to the chosen thresholds for merging variants.

 This project aims to get "trustworthy SV calls across SARS-Cov-2", but there was only very little effort in validation of the SV set detected as far as I can tell. I was honestly quite surprised to see how many deletions that are >20k long you reported as confident. Is it really possible for SARS-CoV-2 to lose more than 2/3 of its genome? Could they represent some SV calling artefacts? One way how to check the quality of called SVs would be compare phylogenetic relationships of the analysed viruses that are probably available for all the sequenced data anyway and verify that the common SVs are shared within monophyletic clusters.

 Finally, the provided data frame with the input data contains various types of libraries. How does SV calling works on RNA-seq data? To be honest, I am not even sure what "RNA-seq" means in the context of RNA viruses, but I do find strange that all the libraries seem to be treated the same. Could some of those huge deletion be simply the selection process of the non-WGS libraries?

 I understand this is a hackathon paper, and therefore it would be unreasonable to ask for more analyses, but I do believe that the wording of this tool should be toned down a lot. I think this is a good first draft for this ambitious pipeline, but loads of work and thought should be given to it before the called SV sets are called trustworthy.

**CleanSV**

 The idea here is to figure out empirical filtering thresholds to minimise false positives on called SVs. This is a great idea and it's desperately needed to have a better community resources to reduce the need for manual curation.

 I am also very sure that these thresholds would have great applicability in other species and germline SV calling. Is there any chance of more general applicability?

 Given authors have decided not to share the filtering thresholds, I think it should be clearly stated early on that this is only a conceptual description of the workflow and results are not provided yet.

*Minor comments*:

 I resume hs37d5 in "Illumina short reads using novoalign hs37d5 HG002 BAM" means it a human data, but I do think it should be explicitly mentioned.

 A bunch of citations are in a different format, something like "(7)" and without actual reference.

**Sniphles**

 Again, a very timely contribution. Phasing of SVs will me more and more important as long read technologies are more and more available even for population genomics studies.

 I found the idea very clear, and sound. I did wonder, however, about the merging step. Technically, SVs from different halplotype need to be merged as if only the SV is homozygous in the alternative allele, therefore I wonder if it is appropriate to lump together everything that is closer than 1000 bases from each other. How many such variants were detected?

 That's kind of related to the only thing I was missing - results. The test case of this method is basically a more confusing repetition of what is already written in methods without any actual numbers of phased SVs. How successful was phasing? How many and how long blocks were phased? If unavailable yet, you should be explicit that it's untested approach.

 Although I have not tried to install the software, I appreciated that the GitHub repository was very neat and the code very well organised.

**Swagg**

 This was another tool with rather difficult text and figure. It is still not very clear to me how using protein graph should help SV calling.

 I was also rather confused about the construction of protein graph. In methods the you write, "Protein graphs are generated using a multiple sequence alignment of the protein", but what is "the protein"? I originally imagined SWAGG would use genome annotation, where the multiple sequence alignment comes into play? I got it lot better from the presentation I found on the GitHub, but I am still not 100% sure how it's done. The presented figure is very pretty, but I did not understand at all what I was supposed to look at.

 I just think it's pity, it does look like a loads of work with a really interesting idea. I would recommend to spend more effort on explanation of the reasoning behind and general clarity of the text.

 I also wondered, is the protein information used for SV calling in the end? If no, why is not the input to your software a vcf file with SVs? Why it's important they are called by GATK and deepVariant? That was another confusing bit for me.

**PanOriginSV**

 A machine learning approach that takes genome variation graph that includes both SNVs and SVs and determines lab of origin. First, I would like to disclaim I am a bit out of my depth reviewing this method, as I never worked with lab strains and genetically modified organisms or thought about the problem of lab attribution.

 I was a bit confused about the benchmarking. The Genetic Engineering Attribution Challenge seem to have plenty of approaches with really successful prediction algorithm, why is the approach compared to a single method (PlasmidHawk) that does not seem to be compared to the other approaches in the challenge? I also wondered why the results were shown in per-cluster basis, not overall accuracy, so they could be comparable to the approaches from the Genetic Engineering Attribution Challenge.

 Perhaps a silly thought, but do you think that machine learning approach is scalable? Having comparable training dataset to the unclassified dataset is essential for meaningful prediction, would the approach work for novel strains?

*Minor comments:*

 This sentence "PanOriginSV has three additional open source dependencies which are MMSEQ2 (for clustering), BCALM (pangenome creation) and GraphAligner (for sequence-to-graph alignment)" should probably contain references to respective software.

 "10-50x less than the linear model (depending on the cluster)." Less than PlasmidHawk?

**GeneVar**

 This tool has a functional prototype and it works! It allows a user to browse variants related to any human gene, which is fun. However, I do think it should be called "human SV browser" (I originally imagined the intend is to make a generic SV browser).

 I appreciated to attempt of making information about human SVs accessible to more general public, but I do wonder how close to the goal authors got. I don't think allele frequency spectrum is an easy plot for a user without background in genetics. Also, there are loads of variants shown for every gene, would it be possible in future to somehow prioritise to the "most likely relevant" variants? What you think could be done to improve accessibility of the tool by for example GPs?

*Minor comment*:

 In the app, it's a bit confusing that SV length is filtered on the panel on left, while all the remaining variables are filtered by clicking on the empty "all" fields bellow column names.

**SVTeaser**

 I really like this idea for a framework for simulating reads and benchmarking SV callers. Beside previously utilised approach of generating insilco generated SVs, SVTeaser also allows a lexicon based generation of SVs. I also found the writing and description of this method very nice and understandable.

 One of the main general problems of simulating sequencing reads is that it is very hard to take into consideration all biases of real life sequencing. For example, cross sample or bacterial contamination are really hard to consider. As a consequence, benchmarking using simulations is (probably) always overly optimistic. Would it be useful to attempt to quantify the optimism? For example by simulating reads from a sequenced genome with known SVs? Then the precision and recall of SVs could be compared between the simulated and real sequencing data.

**XSVLen**

 Sound approach, but took me while to see any added value compared to the original study by Chin *et al.* (2020) ^1^. Do I understand right that what was done on MHC locus you generalised in a framework for any region with haplotype-resolved assemblies? I think it should be more clearly stated what was done by by Chin *et al.* before and how your approach differs. Also, in the text you refer to Nurk *et al.*, although the reference is Chin *et al.* (I think it was confused with this other paper presenting HiCanu ^2^). 

 In the table, the second row says "Number of these calls that overlap assembly contigs". First, how can be any variant called outside of an assembly contig? Do you mean overlapping with haplotype-resolved contigs? You also write "these calls", which made me think it's a subset of the first row, but insertions >50bp seems to have a higher number in the first row (14942 vs 17481). Actually, I did not really understand the third row either, where the "Number of truvari" come from? Are these cuteSV compared to the gold standard? If so, how comes the FP and TP do not sum up to the same number as the first row?

 Similarly to SWAGG, I think this could be a useful tool and you seem to do loads of work on the coding part. However, the text was very confusing to me and I do think with more effort in it you could reach much wider readership.

*Minor comments:*

 This sentence is hard to read "This software is a framework for haplotype-resolved assemblies for benchmarking SV detection algorithms (Figure 10).", what about "This software is a framework for benchmarking SV detection algorithms against haplotype-resolved assemblies (Figure 10)."?

 "All methods are open-source licensed and have been made available on GitHub: https://github.com/collaborativebioinformatics." is an unnecessary statement as FAIR principles are disclosed for all 10 tools and all the links are provided at the bottom.

Are the conclusions about the tool and its performance adequately supported by the findings presented in the article?

Yes

Is the rationale for developing the new software tool clearly explained?

Partly

Is the description of the software tool technically sound?

Partly

Are sufficient details of the code, methods and analysis (if applicable) provided to allow replication of the software development and its use by others?

Yes

Is sufficient information provided to allow interpretation of the expected output datasets and any results generated using the tool?

Yes

Reviewer Expertise:

Genomics, evolutionary biology, bioinfromatics

I confirm that I have read this submission and believe that I have an appropriate level of expertise to confirm that it is of an acceptable scientific standard, however I have significant reservations, as outlined above.

## References

[1] : A diploid assembly-based benchmark for variants in the major histocompatibility complex. *Nature Communications*.2020;11(1) : 10.1038/s41467-020-18564-910.1038/s41467-020-18564-9PMC750883132963235

[2] : HiCanu: accurate assembly of segmental duplications, satellites, and allelic variants from high-fidelity long reads.*Genome Res*.30(9) : 10.1101/gr.263566.1201291-130510.1101/gr.263566.12032801147PMC7545148

# Response

**Published:** 2021-06-06

**Authors:** Ann Mc Cartney

**Affiliations:** NIH, USA

***We would like to thank Kamil Jaron for his time on giving the authors of this manuscript feedback and comments. Upon receiving these comments the corresponding authors reached out to the developers of each of the tools developed as part of this hackathon, each of which have done their utmost to respond to each comment or question. The responses to each of the reviewer's comments are outlined below in bold and italic.***

**Comment 1: General: **This manuscript is a write-up report of an apparently very successful hackathon. Authors present an overview of 10 different approaches to solve various issues in the genomics world of structural variants, some of them sound really promising. I appreciated the modest tone authors have chosen when describing the current state of each tool. All the tools have at least some exploratory value, and all of them are publicly available, therefore I have no major objections for the manuscript to be indexed. This being said, I do agree with the first reviewer, I would greatly appreciate it if before diving into details of individual methods, a big picture overview would be provided, for example a table with short descriptions, urls and development stages would be really handy. Disclosing the development stages would be particularly useful for a reader to know what to expect if proceeding further in reading.I do think this manuscript will spark a lot of ideas in the SV community, so I also wondered, what are the contribution policies to individual tools? Many of the tools have some future plans and are not yet production ready. Who would be the people to contact if someone would be interested in contributing and helping out with finishing a tool that would be particularly helpful for their research project? 

***Response: This is a great question. In general the tools are provided as is. The individual authors are part of their github pages. Furthermore these tools might be extended in future hackathons. Some tools are still being worked on (e.g. NibSV). In terms of contacting authors, please contact the authors associated with each tool; this should be listed in each README of the respective tool. Naturally, feel free to contact the lead authors of this paper as well.***

**Comment 2: General:** The manuscript could benefit immensely from a big reorganization. I do believe that the classical Intro/Meth/Results organization is not doing this manuscript a favor. It was really hard for me to keep track of ten threads in parallel and at some point I just gave up on top-bottom reading. I ended up scrolling back and forth between different sections to get information regarding one tool I tried to understand at the time. I also think most of the people will be mostly interested in one of the tools only when opening the manuscript for reading, therefore I would propose to keep the common Introduction and Conclusion sections but restructure the rest of the paper on a per method basis. 

***Response: While we agree, this is a given from the journal for hackathons. We are following this format.***

**Comment 3: General:** I also appreciated the authors' effort to comply with FAIR principles. Although I have not checked every tool, those I did were neatly documented. Thoughts regarding individual tools are listed below. 

***Response: We thank the reviewer for going over these and highly appreciate the feedback.***

**Comment 4: **For the NibblerSV Developers:If I understand right. The genotyping using NibblerSV is done in two phases - 1. calculating a dictionary of specific kmers for each allele, I would call those diagnostic kmers (e.g. allele specific kmers absent in the reference genome) and 2. matching kmers of resequencing data to the diagnostic kmers. I did find the idea very clearly explained. The part I did not understand is how it's decided if a sample does or does not carry a variant. Does it need to have all the diagnostic kmers at a certain coverage? Or at least some proportion? What if the sample contains kmers of multiple diagnostic markers? Is it possible to genotype a heterozygous SV? Is there any measure of confidence in genotyped alleles? I personally would prefer to read more about these details over technical aspects such as different types of temporary files that the software is producing that actually are mentioned. 

***Response: NibSV currently screens the diagnostic kmers against the reference genome. Right now if one of these kmers are found in the reads it is reported that the SV is found in general. This can be improved upon easily (e.g. weight with reference allele kmers) but that is the current state. NibSV does not include a genotyping model nor ref alleles at the moment. Still the early results are very promising. The tests were done using GIAB Tier 1 v0.6 truth SV set and alignment file of illumina 2x250 bp dataset for HG002 sample . The true positive count is the number of genotyped SVs that are not parental SVs i.e. HG2count > 0 and the FP count is the parental SVs i.e. HG2count=0. The parental SVs that are not genotyped are counted as TNs and the non-parental SVs that are not genotyped by nibSV are counted as FN. The following metrics are used to evaluate performance of NibSV: Recall = TP/ (TP+FN); Precision = TP/(TP+FP); Accuracy = (TP+TN)/(TP+FP+FN+TN); False discovery rate = FP/(TP+FP).We have compared the performance of nibSV with SVTyper (will update the results in the manuscript). The Paragraph tools required to add the padding bases to the SVs in the truth set. We could not successfully run paragraph without making any changes to the truth set. Therefore, we have not included the paragraph tool in this result.***

**Comment 5: **For NibSV Developers: I also wondered, given the approach of generating catalogues of SV-diagnostic kmers, I would anticipate very variable power to detect SVs of different sizes and types. For example, recent inversions will be identified only through very few kmers found at the edge of the inversion, compared to large scale insertions that will probably harbour many diagnostic kmers and clearer signals. Did you look at the sensitivity/precision in respect to size/type? Would that be a consideration one should have when deciding if to use nibSV or paragraph or VG? 

***Response: This is again an excellent point. We only focused on insertion and deletions at this moment. Indeed we see a higher recall on insertion compared to deletions. This is also driven by the fact that we see a deletion more often within repeats. Nevertheless, nibSV does not consider the entire length of an insertion but rather only the breakpoints. We think that nibSV has some advantages over runtime and its not reliant over the genome version of the reads. However, Paragraph etc. will likely have more control of different insertions at the same region.***

**Comment 6:** For the CNV2SV Developers: This tool has the least intuitive motivation of all the tools presented. I would naturally assume all loci with CNV should also be called as SVs, hence I was not sure why it is interesting at all getting the same information twice. However, the results of this analysis proved my intuition wrong. Only very few CNVs are linked to SVs. However, the suggested explanation with length disparity was a bit dissatisfying to me. How come that the SV and CNV callers call variants of so different sizes? Which are shorter/longer? Do you think the CNV/SV reconciliation could be more overlapping with a different choice of SV/CNV caller? This software has an absolutely outstanding description on the GitHub repository. Minor comments: Figure 12, panel B has a cropped y-axis label. In the Methods the text "formats (from Parliament" has an opening bracket that is never closed. 

***Response: Typically CNV is called against a locus on a reference genome. We take the view that a CNV is caused by one or more SVs but most CNV callers do not identify a specific SV (insertions or deletions with the coordinate associated.) For example, if we know there are additional copies for region A. A CNV caller will call an additional copy of A in the reference coordinate, but one would not know where the additional copy really is and what the exact sequences and the inserted locus. CNV2SV is partially developed to understand the spectrum of CNV. By connecting the CNV calls for SV calls from de novo assembly results, we get a chance to understand the nature of CNVs is the light full de novo assembly results. The differences of the sizes from the different CNV caller and SV caller actually reflects that we still need more work to understand the relationship of CNVs and SVs. (Most CNV callers use short read data with coverage statistics. This is fundamentally different from calling SVs from assembly-assembly alignment in terms of detection methodology.) We anticipate more de novo assemblies of human genomes will help us eventually get better pictures.***

**Comment 7:** For the CoronaSV Developers: I did appreciate the idea of a scalable pipeline for conservative analyses of SARS-CoV-2 genomes. Authors made many unjustified choices. For example, why did you choose in particular Manta, Delly, Lumpy and Tardis to detect SV using short reads? But I suppose it is understandable why it would be challenging to justify every choice in the limited time of a hackathon. The step of the analysis I am worried about is the merging by SURVIVOR, it also happens to be the only one that does not seem to be included in the GitHub repository. What parameters were used for individual merging steps using SURVIVOR? Given the genome of SARS-CoV-2 is really small, I believe that the outcome of this pipeline will be very sensitive to the chosen thresholds for merging variants. This project aims to get "trustworthy SV calls across SARS-Cov-2", but there was only very little effort in validation of the SV set detected as far as I can tell. I was honestly quite surprised to see how many deletions that are >20k long you reported as confident. Is it really possible for SARS-CoV-2 to lose more than 2/3 of its genome? Could they represent some SV calling artefacts? One way to check the quality of called SVs would be to compare phylogenetic relationships of the analysed viruses that are probably available for all the sequenced data anyway and verify that the common SVs are shared within monophyletic clusters. Finally, the provided data frame with the input data contains various types of libraries. How does SV calling work on RNA-seq data? To be honest, I am not even sure what "RNA-seq" means in the context of RNA viruses, but I do find it strange that all the libraries seem to be treated the same. Could some of those huge deletions be simply the selection process of the non-WGS libraries? I understand this is a hackathon paper, and therefore it would be unreasonable to ask for more analyses, but I do believe that the wording of this tool should be toned down a lot. I think this is a good first draft for this ambitious pipeline, but loads of work and thought should be given to it before the SV sets are called trustworthy.

***Response: The reviewer makes several helpful points. To the first point, we selected a handful popular SV callers from short reads suitable for the task, and given the short duration of the hackathon, we unfortunately were unable to evaluate a more comprehensive set. To the general point of do we expect to see such large SVs, given SARS-CoV-2 is sequenced from an intrahost population, which often is from metatranscriptomes (RNA-seq applied to a microbiome sample) containing subgenomic RNAs, large SVs can be observed as it captures the transcriptional landscape of SARS-CoV-2. And we have added details of SURVIVOR to the Github repository, thank you for pointing this out.***

**Comment 8: **For the CleanSV Developers: The idea here is to figure out empirical filtering thresholds to minimise false positives on called SVs. This is a great idea and it's desperately needed to have better community resources to reduce the need for manual curation. I am also very sure that these thresholds would have great applicability in other species and germline SV calling. Is there any chance of more general applicability? 

***Response: We thank the reviewer for the assessment and insightful question. In terms of more general applicability, we agree with the reviewer entirely. However, in order to release these filters/thresholds to the larger community for general use, the optimal approach would be to focus on a particular use of SV calling (e.g. translational genomics in oncology) with a particular assay of a certain average coverage and tumor purity. These filters need to be tailored specifically for these parameters. We suspect generating filters per SV caller for general use would result in minimal quality control; this is especially true within cancer genomics in terms of calling somatic and germline SVs accurately. As for focusing on other species (even within a germline context), the main impediment is that we would need a sufficient number of samples from species X at a specific time in order to create these filters.***

*** Comment 9: ***For the CleanSV Developers: Given authors have decided not to share the filtering thresholds, I think it should be clearly stated early on that this is only a conceptual description of the workflow and results are not provided yet. 

***Response: We agree with this assessment, and have tried to clarify this in the manuscript. This paper is the result of a hackathon, and the ideas within are largely proof of concepts which have value outside of a more comprehensive paper.***

**Comment 10:** For the CleanSV Developers: Minor comments: I resume hs37d5 in "Illumina short reads using novoalign hs37d5 HG002 BAM" means it is human data, but I do think it should be explicitly mentioned. A bunch of citations are in a different format, something like "(7)" and without actual reference. 

***Response: We have clarified and fixed these issues in the manuscript.***

**Comment 11:** For the Sniphles Developers: Again, a very timely contribution. Phasing of SVs will be more and more important as long read technologies are more and more available even for population genomics studies. I found the idea very clear, and sound. I did wonder, however, about the merging step. Technically, SVs from different haplotype need to be merged as if only the SV is homozygous in the alternative allele, therefore I wonder if it is appropriate to lump together everything that is closer than 1000 bases from each other. How many such variants were detected? That's kind of related to the only thing I was missing - results. The test case of this method is basically a more confusing repetition of what is already written in methods without any actual numbers of phased SVs. How successful was phasing? How many and how long blocks were phased? If unavailable yet, you should be explicit that it's an untested approach. Although I have not tried to install the software, I appreciated that the GitHub repository was very neat and the code very well organised. 

***Response: We would like to thank the reviewer for the ideas and suggestions. Also, we would like to mention that, this is a prototype of the ideas, and we opened an issue regarding the suggestions. We shall implement a parameter to set the minimum accepted length to identify 2 heterozygote SVs as one.***

 “How many such variants were detected? That's kind of related to the only thing I was missing - results.”

***We used chromosome 20, it contained 652 SVs with minimum 13 to support SVs. 239 SVs are heterozygote we managed to phase 182 of those (76.15%) in 76 phase blocks using the first method. For the second method, while we completed the tool, and it worked as expected, but sadly it did not give the expected results, we still need to work on debugging and enhancing the second method.***

**Comment 12: **For the Sniphles Developers: “I did wonder, however, about the merging step. Technically, SVs from different halplotype need to be merged as if only the SV is homozygous in the alternative allele, therefore I wonder if it is appropriate to lump together everything that is closer than 1000 bases from each other.”

***Response: We agree with the suggestion, we shall implement a parameter that takes as an input the maximum distance to merge heterozygote SVs.***

**Comment 13:** For the Sniphles Developers: “The test case of this method is basically a more confusing repetition of what is already written in methods without any actual numbers of phased SVs.” 

***Response: We changed it accordingly.***

**Comment 14:** For the Sniphles Developers: “How successful was phasing? How many and how long blocks were phased? If unavailable yet, you should be explicit that it's an untested approach.”

***Response: This is a prototype we worked only on chromosome 20 using HiFi data, we still need to develop our method, we updated the manuscript to state that.***

**Comment 15: **For the Swagg Developers: This was another tool with rather difficult text and figure. It is still not very clear to me how using a protein graph should help SV calling. I was also rather confused about the construction of the protein graph. In the methods you write, "Protein graphs are generated using a multiple sequence alignment of the protein", but what is "the protein"? I originally imagined SWAGG would use genome annotation, where the multiple sequence alignment comes into play? I got it a lot better from the presentation I found on GitHub, but I am still not 100% sure how it's done. The presented figure is very pretty, but I did not understand at all what I was supposed to look at. I just think it's a pity, it does look like a load of work with a really interesting idea. I would recommend spending more effort on explanation of the reasoning behind and general clarity of the text. I also wondered, is the protein information used for SV calling in the end? If not, why is not the input to your software a vcf file with SVs? Why is it important that they are called by GATK and deepVariant? That was another confusing bit for me. 

***Response: We would like to thank the reviewer for their comments and questions, specifically with respect to why we should build a variation graph and not just keep variations in a VCF file. Building graphs from amino acid sequences can be used to align new amino acid sequences to these graphs, thereby finding similarities to other sequences. A modified Smith-Waterman algorithm that works for DAGs (Directed Acyclic Graphs) can be used for this job. Due to how proteins work and fold, it is possible to have a gene between two samples from two different genres in bacteria that are very distant (20-30% sequence similarity), however, due to nonsense mutations, the amino acid sequence similarity is higher (50-60%). For example, in Myxobacteria, looking at two different samples from two different genres or families, building a pangenome graph out of the DNA is not really feasible due to the amount of variations between them. However, looking at amino acid sequences, more similarities can be found. Therefore, with building a graph out of the amino acid, paths can be labeled with metadata (certain conserved nodes for example are associated with antibiotic resistance, aligning a new sequence to this graph, can also tell us about this new sequence). This can additionally be accomplished via HMM modeling, but, building a graph adds a visualization element, and with the recent optimizations in graphical mapping algorithms, it may be faster at aligning a sequence to a graph than using some HMM model. Building on the idea that we can add metadata to paths in the graph, looking at this paper for example, "Jaillard et al. (2018). “ A Fast and Agnostic Method for Bacterial Genome-Wide Association Studies: Bridging the Gap between K-Mers and Genetic Events.” We see how from looking at the graphs, certain nodes can be associated with resistance. However, for example, this method breaks down when the similarity between the sequence we're looking at drops, as it's hard to find shared k-mers then. However, going to amino acid space, the similarity increase and similar methods and algorithms can then be used but with the amino acid alphabet.***

**Comment 16:** For the PanOriginSV Developers: A machine learning approach that takes a genome variation graph that includes both SNVs and SVs and determines lab of origin. First, I would like to disclaim I am a bit out of my depth reviewing this method, as I never worked with lab strains and genetically modified organisms or thought about the problem of lab attribution. I was a bit confused about the benchmarking. The Genetic Engineering Attribution Challenge seems to have plenty of approaches with really successful prediction algorithms, why is the approach compared to a single method (PlasmidHawk) that does not seem to be compared to the other approaches in the challenge? I also wondered why the results were shown on a per-cluster basis, not overall accuracy, so they could be comparable to the approaches from the Genetic Engineering Attribution Challenge. Perhaps a silly thought, but do you think that machine learning approach is scalable? Having a comparable training dataset to the unclassified dataset is essential for meaningful prediction, would the approach work for novel strains? Minor comments: This sentence "PanOriginSV has three additional open source dependencies which are MMSEQ2 (for clustering), BCALM (pangenome creation) and GraphAligner (for sequence-to-graph alignment)" should probably contain references to respective software. "10-50x less than the linear model (depending on the cluster)." Less than PlasmidHawk?

***Response: We’d first like to thank the reviewer for their feedback and questions. While other tools have been developed for the same problem, Plasmidhawk was recently shown to outperform the other state-of-the-art methods while also being more straightforward to run. Additionally, Plasmidhawk does not require a GPU in order to train efficiently. The main reason for showing per-cluster accuracy was to show the performance of our method on large groups of highly similar sequences. In addition, machine learning methods are not well suited for small cluster scenarios due to the lack of training data. Unfortunately, the code for all other methods in the GEAC was unavailable, therefore we were unable to compare results without submitting to the challenge. However, we do think that machine learning is scalable, although the preprocessing of the data will become more important as the dataset grows. For novel strains that have no similarity to the database, our method would not work but we are confident that no other method would. However, for novel strains that have “signatures” in the database, these would be represented by nodes in the pangenome that are relatively unique to certain labs.***

**Comment 17: **For the GeneVar Developers: This tool has a functional prototype and it works! It allows a user to browse variants related to any human gene, which is fun. However, I do think it should be called a "human SV browser" (I originally imagined the intent is to make a generic SV browser). I appreciated the attempt to make information about human SVs accessible to the general public, but I do wonder how close to the goal the authors got. I don't think the allele frequency spectrum is an easy plot for a user without a background in genetics. Also, there are loads of variants shown for every gene, would it be possible in future to somehow prioritise to the "most likely relevant" variants? What you think could be done to improve accessibility of the tool by for example GPs? Minor comment: In the app, it's a bit confusing that SV length is filtered on the panel on the left, while all the remaining variables are filtered by clicking on the empty "all" fields below column names.

***Response: Thank you reviewer for your thoughts and questions. We agree that ranking the variants shown could be helpful for genes with a lot of variants. While right now a long list of variants suggest more filtering needed by the user, it would be better to have the most impactful SVs shown first by default. In terms of improving accessibility of the tool to GPs, etc. We think adding documentation and tutorials to the tool would be helpful, for example to interpret the frequency histogram, and will be added as the tool matures. We are also keen on receiving feedback directly from clinicians and GPs. We are exploring ways to reach out to them to learn what should be changed, improved, or added.***

**Comment 18: **From the SVTeaser Developers: I really like this idea for a framework for simulating reads and benchmarking SV callers. Beside the previously utilised approach of generating insilco generated SVs, SVTeaser also allows a lexicon based generation of SVs. I also found the writing and description of this method very nice and understandable. One of the main general problems of simulating sequencing reads is that it is very hard to take into consideration all biases of real life sequencing. For example, cross sample or bacterial contamination are really hard to consider. As a consequence, benchmarking using simulations is (probably) always overly optimistic. Would it be useful to attempt to quantify optimism? For example by simulating reads from a sequenced genome with known SVs? Then the precision and recall of SVs could be compared between the simulated and real sequencing data. 

***Response: We agree that benchmarking against simulated data is overly optimistic and that it’s hard to accurately replicate library preparation errors such as what you have described. A primary goal of SVTeaser is to assist developers of SV calling algorithms with assessing how their tool would perform without needing to quantify their algorithm’s problems versus their sequencing’s problems. A well benchmarked, production-ready tool shouldn’t rely on only SVTeaser and would always have real data to test against. However, should someone want to simulate more realistic sequencing experiments by adding batch effects such as cross-sample contamination, it would be possible for them to run SVTeaser twice - once over their target sample’s SVs at e.g. 28x coverage and again with a second sample’s SVs at e.g. 2x coverage. Then, simply concatenating the sets of generated reads prior to read-mapping would create a simplistic simulation of ~5% cross-sample contamination. While SVTeaser doesn’t currently automate this kind of operation, we will strongly consider how best to incorporate the functionality.***

**Comment 19:** For the developers of XSVLen: Sound approach, but took me a while to see any added value compared to the original study by Chin et al. (2020) 1. Do I understand right that what was done on MHC locus you generalised in a framework for any region with haplotype-resolved assemblies? I think it should be more clearly stated what was done by Chin et al. before and how your approach differs. Also, in the text you refer to Nurk et al., although the reference is Chin et al. (I think it was confused with this other paper presenting HiCanu 2). In the table, the second row says "Number of these calls that overlap assembly contigs". First, how can there be any variant called outside of an assembly contig? Do you mean overlapping with haplotype-resolved contigs? You also write "these calls", which makes me think it's a subset of the first row, but insertions >50bp seems to have a higher number in the first row (14942 vs 17481). Actually, I did not really understand the third row either, where does the "Number of truvari" come from? Are these cuteSV compared to the gold standard? If so, how come the FP and TP do not sum up to the same number as the first row? Similarly to SWAGG, I think this could be a useful tool and you seem to do loads of work on the coding part. However, the text was very confusing to me and I do think with more effort in it you could reach much wider readership.Minor comments: This sentence is hard to read "This software is a framework for haplotype-resolved assemblies for benchmarking SV detection algorithms (Figure 10).", what about "This software is a framework for benchmarking SV detection algorithms against haplotype-resolved assemblies (Figure 10)." 

***Response: Many thanks for the comments and all the interest about the work we performed. The assembly we used for benchmarking is the one generated by Wenger et al. 2020 (NCBI Assembly with accessions GCA_004796485.2 (maternal) and GCA_004796285.1 (paternal) - so we worked with the whole-genome and not only CHM. The reference of CHM locus has been removed from the text and we included the right reference. We have updated the methods section for improving comprehension on the approach we implement. Here, we benchmarked sequence-resolved variants by checking their presence in a haplotype-resolved assembly available and so it works on top of an haplotype-resolved assembly. We have also updated the main table to include the correct numbers and also to help comprehension of categories. The "Number of these calls that overlap assembly contigs" row makes reference to SV called by cuteSV (sequence-resolved SV calling) that would be found also in the assembly by the approach we tested (construct SV query and map queries back to assembly). In the "Number of truvari” (updated to Number of overlapping calls classified with truvari) row we include the classification obtained using truvari – we compare the cuteSV calls and GIAB truth set). This was due to a bug in the annotation script that has been fixed. The minor suggested change has been applied to the text.***

***Again, we thank the reviewer for his thoughtful comments, suggestions and feedback and hope that the following responses have clarified any queries or concerns.***

# Peer review: Reviewer response for version 1

**DOI:** 10.5256/f1000research.54651.r83791

**Published:** 2021-05-11

**Authors:** Francois Sabot

**Affiliations:** 1 DIADE, Univ Montpellier, IRD, CIRAD, Montpellier, France

The Pangenome Hackathon paper lead by A. McCartney and FJ Sedlazeck was a great moment of intellectual development and findings for pangenomics research on human and coronavirus, pandemics time "oblige". Ten softwares/methods were developed, some being even released, some being more in alpha state. All these tools are of interest, in their targeted human-related field but also in non-human fields (working on plants, I am interested in more than one of them).

 The paper has been written collectively, each software participants writing their part, and while of high interest in terms of opening, it is the main limit in the manuscript, in my opinion.

 Indeed there is no homogeneity in the way tools are described (global aim, method, implementation, figure, results/tests), and thus it is quite difficult to have a global idea of the different level of development. An initial table with level of advancement (alpha, beta, ready for use, in testing, etc or something like it) would be useful. In addition, a final reformatting by a single writer would smooth the reading.

 In details, going for each tools:

**NibbleSV**: what is the impact of SNP on the detection through k-mers? I mean if the individual has more than a very low level of variation, it may increase the FP rate? what is the RAM usage per sample? why providing info of time for 23mers but results of recall/precision with 21.

**CNV2SV**: page 8, output of CNV2SV is said to be a python script (envlink.py)? Why computing the pre-alignment on DNAnexus? Which stats methods are used?

**CORONASV**: are the three approaches (SR, LR, assembly) required in all analyses or you can choose? Why does Flye not represented in Figure 3?

**CleanSV**: on page 6, I cannot see the link with aneuploidy in this specific case. Can you estimate the false negative rate? Do you have an idea of the RAM usage?

**Sniphles**: "the produced BAM from the previous step" is the one produced by Princess? The first sentence of results is the same as in the method. It missed also here a short summary of the results.

**Swagg**: the implementation text is almost the same as in the introduction of the tool. The implementation part is quite short and the fig 6 did not shown any info about the protein graph possibility. Finally, what is the advantage of having the protein graph compared to an alignment showing the variation?

**PanOriginSV**: What are the alignment information included in the training part? Which 'more recent pangenome graph construction tool' are you thinking about?

**GeneVar**: a really interesting tool for praticians. My only question is will it be possible to add new informations about variations outside of strandard DB?

**SVteaser**: do you have any information of time per SV? What are the size of variations you can include? Can we add also translocations or SNP?

**XSVLen**: the figure 10 is quite drafty and would need to be improved for more information. Do you have any information about the recall/precision?

 In conclusion, I was very impressed by the high number of tools and of their quality and think this paper is really worthy.

Are the conclusions about the tool and its performance adequately supported by the findings presented in the article?

Partly

Is the rationale for developing the new software tool clearly explained?

Yes

Is the description of the software tool technically sound?

Partly

Are sufficient details of the code, methods and analysis (if applicable) provided to allow replication of the software development and its use by others?

Partly

Is sufficient information provided to allow interpretation of the expected output datasets and any results generated using the tool?

Partly

Reviewer Expertise:

Genomics, bioinformatics, evolution

I confirm that I have read this submission and believe that I have an appropriate level of expertise to confirm that it is of an acceptable scientific standard, however I have significant reservations, as outlined above.

# Response

**Published:** 2021-06-14

**Authors:** Ann Mc Cartney

**Affiliations:** NIH, USA

We would like to thank Francois Sabot for their time on giving the authors of this manuscript feedback and comments. Upon receiving these comments the corresponding authors reached out to the developers of each of the tools developed as part of this hackathon, each of which have highly appreciated your work and thoughtful comments. The responses to each of the reviewers comments are outlined below in bold and italic. 

**General Comment 1: **The Pangenome Hackathon paper led by A. McCartney and FJ Sedlazeck was a great moment of intellectual development and findings for pangenomics research on human and coronavirus, pandemics time "oblige". Ten softwares/methods were developed, some being even released, some being more in alpha state. All these tools are of interest, in their targeted human-related field but also in non-human fields (working on plants, I am interested in more than one of them). The paper has been written collectively, each software participant writing their part, and while of high interest in terms of opening, it is the main limit in the manuscript, in my opinion. Indeed there is no homogeneity in the way tools are described (global aim, method, implementation, figure, results/tests), and thus it is quite difficult to have a global idea of the different levels of development. An initial table with level of advancement (alpha, beta, ready for use, in testing, etc or something like it) would be useful. In addition, a final reformatting by a single writer would smooth the reading. 

***Response: We thank the reviewer for this suggestion. Indeed we also struggled as this manuscript has over 50 authors. The tools have so far only been developed within a few days in the context of a remote hackathon and thus represent more prototypes / proof of concepts than production ready methods.***

**Comment 2: **For NibbleSV Developers: “what is the impact of SNP on the detection through k-mers? I mean if the individual has more than a very low level of variation, it may increase the FP rate? What is the RAM usage per sample? why providing info of time for 23mers but results of recall/precision with 21. 

***Response: That's a great question. We predict that the impact of SNP is minimal because the SNV must be directly within +/- e.g. 10bp of the SV breakpoint on either side. If there is a SNV it will lower our recall rate. We have further experimented with spaced kmers, that would help in this regard. RAM usage is ~4Gbp per sample.***

**Comment 3:** For CNV2SV Developers: page 8, output of CNV2SV is said to be a python script (envlink.py)? Why computing the pre-alignment on DNAnexus? Which stats methods are used? 

***Response: Thank you for your question. The CNV2SV script performs the CNV-SV linking steps independently of the tools used to generate the three input files (2 .vcf files containing SV calls and CNV calls, respectively, as well as the reference genome as .fasta). In the scope of the Hackathon, we generated the test input VCF files using dipcall (genome-genome-alignment for SVs) and CNVnator (a CNV caller), for a human data set. These steps were executed on DNANexus, since unlike the actual CNV2SV script itself, high processing power and working memory were required, especially for the genome-genome alignment for the two human genome versions. Users can use VCF files as generated by tools and parameters of their choice as input for CNV2SV. While we expect output from most relevant tools that produce calls in VCF format to be compatible, we specifically tested the before mentioned tools in the scope of the Hackathon. While the statistics applied to generate the input SV/CNV calls vary based on the tools used, CNV2SV itself performs the linking using default cutoff values for parameters such as minimum CNV length, minimum alignment identity and maximum distance for adjacent duplication events. Furthermore, basic statistics are recorded for matched CNV-SV pairs as well as the failure reasons for CNVs that could not be linked to a corresponding SV. In response to the final question "Which stats methods are used?" the developers were not sure about which stat this is in reference to.***

**Comment 4:** For the CORONASV Developers: are the three approaches (SR, LR, assembly) required in all analyses or you can choose? Why is Flye not represented in Figure 3? 

***Response: We thank the reviewer for catching this; we have updated the text to indicate that long read assembly with Flye is not currently supported for such a small genome with default parameters. Specifically, we have modified "In order to integrate assembly based methods, de novo SARS-CoV-2 assemblies were generated using Unicycler for short-read sequencing and Flye for ONT long-reads." to instead read " In order to integrate assembly based methods, de novo SARS-CoV-2 assemblies were generated using Unicycler for short-read sequencing".***

**Comment 5: **For the CleanSV Developers: Can you estimate the false negative rate? Do you have an idea of the RAM usage? 

***Response: Here we refer to the presence of somatic structural variations to be synonymous with “aneuploidy”. Aneuploidy itself is an umbrella term used within oncology to refer to somatic abnormalities of a cell’s chromosomes. We try to clarify this with a sentence in the manuscript. In terms of the false negative rate, so far this has been estimated with simulated benchmarks, which surely don’t reflect the complexities of actual clinical data---any real data will be of variable tumor content (purity) and coverage. The best source of collective knowledge within the field is probably the results of DREAM challenges. Indeed, the question of false negatives is one of the deeper questions for all variant calling methods used within cancer genomics. Systematically investigating this problem would require benchmarks from real data, which don’t exist---even robustly characterized cell lines would not be realistic enough. With that in mind, we also wish to emphasize that clinicians are primarily concerned with the presence of false positives. These are potential therapeutic biomarkers which, if missed/ignored by bioinformatic methods, could result in overlooked therapeutic interventions. In terms of RAM usage, the tool provided uses standard functions in R, and will not be memory intensive.***

**Comment 6:** For the Sniphles Developers: "the produced BAM from the previous step" is the one produced by Princess? The first sentence of results is the same as in the method. I also missed a short summary of the results. 

***Response: We would like to thank the reviewer, a change was made accordingly in the manuscript.***

**Comment 7:** For the Swagg Developers: the implementation text is almost the same as in the introduction of the tool. The implementation part is quite short and the fig 6 did not show any info about the protein graph possibility. Finally, what is the advantage of having the protein graph compared to an alignment showing the variation? 

***Response: Generating the graphs out of the amino acid has a visualization factor, as different sequences can be represented as different paths in the graph, conserved sequences in the MSA will show up as a single node and bubbles are generated from the variations. One can extend this be aligning new sequences to the graph, e.g. using a modified Smith-Waterman algorithm after topologically sorting the graph, this way, the alignment can tell us quickly which path this new sequence takes through this graph, moreover, one can add another layer of data to the paths, for example in bacteria, certain mutations or conserved sequences in the protein are related to antibiotic resistance.***

**Comment 8:** For the PanOriginSV Developers: What is the alignment information included in the training part? Which 'more recent pangenome graph construction tool' are you thinking about? 

***Response: We’d first like to thank the reviewer for their questions. We thread/align the query sequences to the pangenome graph created using BCALM. The nodes that are hit are used as the features for machine learning. We have experimented with other information as well such as alignment length, %id, etc, but overfitting is an issue that requires further work. Minigraph would be a more recent pangenome graph construction tool that we could use.***

**Comment 9:** For the GeneVar Developers: a really interesting tool for practitioners. My only question is will it be possible to add new information about variations outside of standard DB? 

***Response: Thank you reviewer, for the question. At this time, we don’t plan to support non-standard data types. However, if you have a specific use case in mind, the developers would be open to a collaboration.***

**Comment 10:** For the SVteaser Developers: do you have any information of time per SV? What are the sizes of variations you can include? Can we also add translocations or SNP? 

***Response: Thank you to the reviewer for the feedback. In terms of running time per SV, this correlates with the size of SV simulated and the nature of the SV (e.g. translocations being more difficult). However, these operations are relatively straightforward operations manipulating the strings from the FASTA file. Users are able to set the size of SVs within the simulation framework, and in principle could handle any organism. Yes, translocations and SNPs are possible.***

**Comment 11: **For the XSVLen Developers: the figure 10 is quite drafty and would need to be improved for more information. Do you have any information about the recall/precision? 

***Response: Many thanks for the suggestion and the interest, the figure is intended to give an overview of the method and not a detailed explanation of the pipeline. Regarding precision/recall numbers, we did not check this specifically but in Table 2 the number of TP and FP are summarized for the assembly calls.***
